# Wave Based Method for Free Vibration Analysis of Cross-Ply Composite Laminated Shallow Shells with General Boundary Conditions

**DOI:** 10.3390/ma12233808

**Published:** 2019-11-20

**Authors:** Dongyan Shi, Dongze He, Qingshan Wang, Chunlong Ma, Haisheng Shu

**Affiliations:** 1 College of Mechanical and Electrical Engineering, Harbin Engineering University, Harbin 150001, China; shidongyan@hrbeu.edu.cn (D.S.); Hdz2012071506@126.com (D.H.); shuhaisheng@hrbeu.edu.cn (H.S.); 2 State Key Laboratory of High Performance Complex Manufacturing, Central South University, Changsha 410083, China; 3 Department of Automotive Engineering, Harbin Vocational & Technical College, Harbin 150001, China; machunlong@hrbeu.edu.cn

**Keywords:** composite laminated shallow shell, free vibration characteristics, classical and elastic boundary conditions, general boundary conditions

## Abstract

In this paper, a semi-analytical method is adopted to analyze the free vibration characteristics of composite laminated shallow shells under general boundary conditions. Combining two kinds of shell theory, that is, first-order shear deformation shell theory (FSDT) and classical shell theory (CST), to describe the dynamic relationship between the displacement resultants and force vectors, the theoretical formulations are established. According to the presented work, the displacement and transverse rotational variables are transformed into wave function forms to satisfy the theoretical formulation. Related to diverse boundary conditions, the total matrix of the composite shallow shell can be established. Searching the determinant of the total matrix using the dichotomy method, the natural frequency of composite laminated shallow shells is obtained. Through several classical numerical examples, it is proven that the results calculated by the presented method are more accurate and reliable. Furthermore, to discuss the effect of geometric parameters and material constants on the natural frequencies of composite laminated shallow shells, some numerical examples are calculated to analyze. Also, the influence of boundary elastic restrained stiffness is discussed.

## 1. Introduction

The shallow shell is an open shell with a small curvature and radius of curvature compared with various shell parameters (i.e., length and width). With the development research into composite materials, composite laminated shallow shells are widely applied in some modern engineering practice with a high level of intensity and rigidity, for instance, petroleum equipment, aerospace equipment, and marine equipment. It is worth noting that the composite laminated shallow shells are typically operated under complicated environmental conditions and subjected to complex boundary conditions. So, it is particular importance to fully investigate the free vibration characteristics of composite laminated shallow shells with non-classical boundary conditions.

Through many years of hard work by research scholars, some shell theories have been summarized, such as classical shell theory (CST) [1,2,3], first-order shear deformation shell theory (FSDT) [4,5], and high-order shell theory (HST) [6,7,8,9,10]. CST is the basic shell theory and is known as the simplest equivalent single layer, which is based on the Kirchhoff–Love hypothesis. To analyze the complex shell structure, some shell theories were developed along with some assumptions, such as Reissner–Naghdi’s shell theory and Donner–Mushtari’s theory. A more detailed description of these theories can be found in the research by Reddy [11], Leissa [12], and Qatu [13]. The main application area is thin shell structures. To analyze the thick shell, CST ignores the effect of transverse shear deflection, causing the calculation of natural frequencies to be inaccurate. To improve the influential impact of transverse shear deformation, FSDT is conducted. HST can attenuate the dependence of FSDT on shear correction factors; however, there is a large amount of calculation in the study of the high-order stress resultant force. Simultaneously, many remarkable researchers have investigated the composite laminated shallow shell in recent years and published some excellent papers. Ye et al. [14] investigated the free vibration characteristics of the composite laminated shallow shell under general elastic boundary conditions. The closed form auxiliary functions are used to transform the displacement variables into standard Fourier cosine series. Kurpa et al. [15] extended the R-function method to investigate the composite laminated shallow shells on an arbitrary planform by FSDT. Fazzolari and Carrera E [16] conducted the Ritz formulation and Carrera unified formulation to investigate the composite laminated doubly-curved anisotropic shell, and the free vibration response is discussed. Awrejcewicz et al. [17] proposed R-functions theory and the spline-approximation to study the bending performance of the composite shallow shell with a static loading boundary condition. Tran et al. [18] presented a static feature of the cross-ply composite hyperbolic shell panels on Winkler–Pasternak elastic foundation, and the smeared stiffeners technique was adopted. Biswal et al. [19] discussed the free vibration characteristic of composite shells consisting of woven fiber glass/epoxy with hygrothermal environments. The FSDT and quadratic eight-noded isoparametric element are adopted to study the free vibration characteristics under elevated temperatures and moisture concentrations conditions. Garcia et al. [20] investigated the effect of polycaprolactone nanofibers on the dynamic behavior of glass fiber reinforced polymer composites. Garcia et al. [21] investigated the influence of the inclusion of nylon nanofibers on the global dynamic behaviour of glass fibre reinforced polymer (GFRP)composite laminates. Shao et al. [22] conducted the enhanced reverberation-ray matrix (ERRM) method to investigate the transient response of the composite shallow shell. In these studies, the kinetic analysis of composite laminated shallow shell is proposed to free vibration, and many analytical and computational methods were developed.

These include the Ritz method [23,24,25,26,27], dynamic stiffness method [28], closed-form solution [29,30,31], boundary domain element method [32], Meshfree approach [33], Galerkin method [34,35], and finite element method [36,37,38].

In recent years, the wave-based method (WBM) has been adopted to investigate the dynamic behavior of engineering structures in some applications. WBM was first proposed in the work of [39] to analyze the coupled vibro-acoustic systems and the steady-state dynamics characteristics of the system concerned. Deckers et al. [40] presented a literature review of WBM research for 15 years. With the research on structural vibration in recent years, WBM has been adopted in the dynamic analysis for some engineering structures, such as the dynamic characteristics of cylindrical shell structures, which many researchers have studied using WBM. Chen et al. [41] analyzed the free and force vibration characteristics of a cylindrical shell in discontinuity thickness form. Xie et al. [42] conducted WBM to study the free vibration and acoustic dynamic characteristics of underwater cylindrical shells with bulkheads. Wei et al. [43] investigated the non-uniform stiffener distribution of a cylindrical shell. At the same time, as many reinforcements and coupling structures are more common in engineering applications, the corresponding research is increasing, such as the cylindrical shell coupled elastically with annular plate and the ring stiffened cylindrical shell with frame ribs [44,45]. Also, the free vibration characteristics of the composite laminated cylindrical shell have been investigated [46]. Therefore, it is meaningful to develop an effective method for the general processing ability of composite laminated shallow shells with general boundary conditions. According to the author’s literature review of related topics, there has not been any published work with regard to the application of the presented method to analyze the free vibration characteristics of the composite laminated shallow shell with general boundary conditions.

For the first time, the wave-based method is adopted to study the free vibration characteristics for a composite laminated shallow shell with general boundary conditions. According to the relationship between the displacement vector and force resultants, the governing equation of composite shells is established by FSDT and CST. By converting the displacement variable into a wave function form and the boundary matrices, the total matrix is established. Solving the root of the total matrix determinant using the dichotomy method, the natural frequencies of composite laminated shallow shells are calculated. To verify the correctness of the solutions by the presented method, the comparisons of the current solutions with the results in represented literatures are shown. Furthermore, the influence of material parameters and geometric constants, such as length to radius ratios, length to thickness ratios, modulus ratios, and elastic restrained constants, are discussed in some numerical examples. The main purpose of this paper is to provide a relatively new method for analyzing the free vibration characteristics of composite laminated shallow shells, which provides a new direction for composite laminated structure analysis. When studying the vibration analysis of the composite laminated shallow shell with general boundary conditions, it is easier to obtain the total matrix, and the boundary conditions are easy to replace. The advantages of the presented method lie in its simplicity, low computational cost, and high precision.

## 2. Theoretical Formulations

### 2.1. The Description of Model

**Figure 1:**
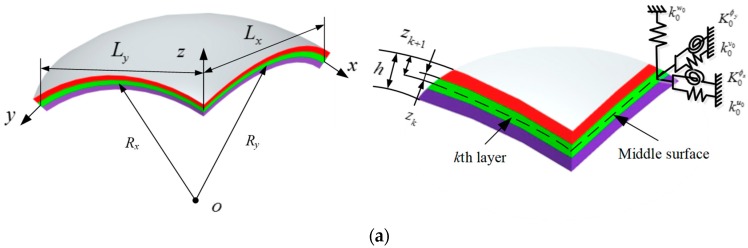

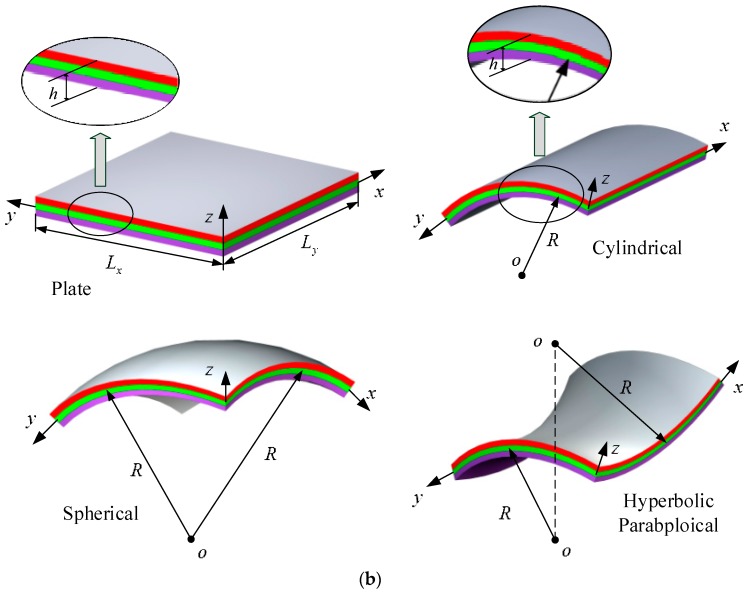
(**a**): Geometric model of the composite laminated shallow shell with elastic restraint; (**b**): geometric model of the composite laminated shallow shell with various curvature types.

In Figure 1a, the schematic diagrams of the composite laminated shallow shells under elastic restraint are shown. *L_x_*, *L_y_*, and *h* express the length, width, and thickness, respectively, of the composite laminated shallow shells. *R_x_* and *R_y_* indicated the principle curvature radii. In the middle surface of the model, a global coordinate (*o*-*xyz*) is established in the length, width, and thickness directions. For the *k*th layer of the composite shell, the distances of top and bottom surface to the middle surface are denoted as *Z_k_*_+1_ and *Z_k_*. For the elastic boundary conditions, there is one set of linear springs (*K_u_*, *K_v_*, and *K_w_*) and one pair of rotational springs (*K_ϕx_* and *K_ϕy_*), which set on two edges, *x* = 0 and *L_x_*. Through the changing of two pairs of elastic restrained springs, an arbitrary elastic boundary condition can be achieved. In Figure 1b, with the changing of the principle curvature radii, the composite laminated shallow shells have various types, such as plate (i.e., *R_x_* = *R_y_* = ∞), cylindrical shell (i.e., *R_x_* = *R*, *R_y_* = ∞), spherical shell (i.e., *R_x_* = *R_y_* = *R*), and hyperbolic paraboloidal shell (i.e., *R_x_* = −*R_y_* = *R*).

### 2.2. First-Order Shear Deformation Shell Theory (FSDT)

#### 2.2.1. Kinematic Relations and Stress Resultants

This section is divided into subheadings. It should provide a concise and precise description of the experimental results and their interpretation, as well as the experimental conclusions that can be drawn.

According to the relationship between the displacement variables and rotation transverses of the composite shallow shells by FSDT, the displacement variables are shown as follows [13]:

(1) $$\begin{array}{l} u\left(x,z,t\right)=u_{0}\left(x,t\right)+z\phi_{x}\left(x,t\right)\\ v\left(x,y,t\right)=v_{0}\left(x,y\right)+z\phi_{y}\left(x,y\right)\\ w\left(x,z,t\right)=w\left(x,t\right) \end{array}$$

where *u*_0_, *v*_0_, and *w*_0_ are the displacements of the arbitrary point along the *x*, *y*, and *z* directions, respectively, in the middle surface. *ϕ_x_* and *ϕ_y_* are the *y* and *x* axes transverse rotations, respectively, and *t* is a time variable. The linear strain relationship between the change strain and curvature in the middle surface under the assumption of small deformation is given as follows:

(2) $$\begin{array}{l} \varepsilon_{xx}=\varepsilon_{xx}^{0}+z\varepsilon_{xx}^{1}\\ \varepsilon_{yy}=\varepsilon_{yy}^{0}+z\varepsilon_{yy}^{1}\\ \gamma_{xy}=\gamma_{xy}^{0}+z\gamma_{xy}^{1}\\ \gamma_{xz}=\gamma_{xz}^{0}\\ \gamma_{yz}=\gamma_{yz}^{0} \end{array}$$

where {$\varepsilon_{xx}^{0}$, $\varepsilon_{yy}^{0}$} are the normal strains of the middle surface, {$\gamma_{xy}^{0}$, $\gamma_{xz}^{0}$, $\gamma_{yz}^{0}$} are the shear stains, and {$\varepsilon_{xx}^{1}$, $\varepsilon_{yy}^{1}$, $\gamma_{xy}^{1}$} are the curvature and twisting changes of the middle surface. The detailed expressed formulations of the strains and changes are defined as follows:

(3) $$\begin{array}{ll} \varepsilon_{xx}^{0}=\frac{\partial u_{0}}{\partial x}+\frac{w_{0}}{R_{x}} & \varepsilon_{yy}^{0}=\frac{\partial v_{0}}{\partial y}+\frac{w_{0}}{R_{y}}\\ \varepsilon_{xx}^{1}=\frac{\partial \phi_{x}}{\partial x} & \varepsilon_{yy}^{1}=\frac{\partial \phi_{y}}{\partial y}\\ \gamma_{xy}^{0}=\frac{\partial v_{0}}{\partial x}+\frac{\partial u_{0}}{\partial y} & \gamma_{xy}^{1}=\frac{\partial \phi_{y}}{\partial x}+\frac{\partial \phi_{x}}{\partial y}\\ \gamma_{xz}^{0}=\frac{\partial w_{0}}{\partial x}-\frac{u_{0}}{R_{x}}+\phi_{x} & \gamma_{yz}^{0}=\frac{\partial w_{0}}{\partial y}-\frac{v_{0}}{R_{y}}+\phi_{y} \end{array}$$

The corresponding stresses expressed by the Hooke’s law are as follows:

(4) $$\left\{\begin{array}{l} \sigma_{xx}\\ \sigma_{yy}\\ \tau_{xy}\\ \tau_{xz}\\ \tau_{yz} \end{array}\right\}=\left[\begin{array}{ccccc} \bar{Q_{11}} & \bar{Q_{12}} & 0 & 0 & \bar{Q_{16}}\\ \bar{Q_{12}} & \bar{Q_{22}} & 0 & 0 & \bar{Q_{26}}\\ 0 & 0 & \bar{Q_{44}} & \bar{Q_{45}} & 0\\ 0 & 0 & \bar{Q_{45}} & \bar{Q_{55}} & 0\\ \bar{Q_{16}} & \bar{Q_{26}} & 0 & 0 & \bar{Q_{66}} \end{array}\right]\left\{\begin{array}{l} \varepsilon_{xx}\\ \varepsilon_{yy}\\ \gamma_{xy}\\ \gamma_{xz}\\ \gamma_{yz} \end{array}\right\}$$

where $\bar{Q_{ij}}$ (*i*, *j* = 1,2,4,5,6) are the transform coefficients and depend on material parameters; and the constants *Q_ij_* (*i*,*j* = 1,2,4,5,6), which are associated with the strains and stresses, can be expressed as follows:

(5) $$\begin{array}{c} Q_{11}=\frac{E_{1}}{1-\mu_{12}\mu_{21}},Q_{12}=Q_{21}=\frac{\mu_{12}E_{2}}{1-\mu_{12}\mu_{21}},Q_{22}=\frac{E_{2}}{1-\mu_{12}\mu_{21}}\\ Q_{44}=G_{23},Q_{55}=G_{13},Q_{66}=G_{12} \end{array}$$

where *E*_1_, *E*_2_ are the Yong’s moduli and *μ*_12_ and *μ*_21_ are the Poisson’s ratios. By integrating the stresses and moments over the cross section and thickness, the relationship between the strains and curvature in the middle surface is given as follows:

(6) $$\left\{\begin{array}{c} N_{xx}\\ N_{yy}\\ N_{xy}\\ M_{xx}\\ M_{yy}\\ M_{xy} \end{array}\right\}=\left[\begin{array}{cccccc} A_{11} & A_{12} & A_{16} & B_{11} & B_{12} & B_{16}\\ A_{12} & A_{22} & A_{26} & B_{12} & B_{22} & B_{26}\\ A_{16} & A_{26} & A_{66} & B_{16} & B_{26} & B_{66}\\ B_{11} & B_{12} & B_{16} & D_{11} & D_{12} & D_{16}\\ B_{12} & B_{22} & B_{26} & D_{12} & D_{22} & D_{26}\\ B_{16} & B_{26} & B_{66} & D_{16} & D_{26} & D_{66} \end{array}\right]\left\{\begin{array}{c} \varepsilon_{xx}^{0}\\ \varepsilon_{yy}^{0}\\ \gamma_{xy}^{0}\\ \varepsilon_{xx}^{1}\\ \varepsilon_{yy}^{1}\\ \gamma_{xy}^{1} \end{array}\right\},\left\{\begin{array}{c} Q_{y}\\ Q_{x} \end{array}\right\}=K_{c}\left[\begin{array}{cc} A_{44} & A_{45}\\ A_{45} & A_{55} \end{array}\right]\left\{\begin{array}{c} \gamma_{yz}^{0}\\ \gamma_{xz}^{0} \end{array}\right\}$$

where {*N_xx_*, *N_xy_*, *N_yy_*} is the in-plane force resultant, {*M_xx_*, *M_yy_*, *M_xy_*} is the bending and twisting moment resultant, and {*Q_x_*, *Q_y_*} is the transverse shear force resultant. *K_c_* is the shear correction factor and the value is set as 5/6. Furthermore, the stretching stiffness coefficients, coupling stiffness coefficients, and bending stiffness coefficients are given as follows:

(7) $$A_{ij}=\sum_{k=1}^{N}\bar{Q_{ij}}\left(Z_{k+1}-Z_{k}\right),B_{ij}=\frac{1}{2}\sum_{k=1}^{N}\bar{Q_{ij}}\left(Z_{k+1}^{2}-Z_{k}^{2}\right),D_{ij}=\frac{1}{3}\sum_{k=1}^{N}\bar{Q_{ij}}\left(Z_{k+1}^{3}-Z_{k}^{3}\right)$$

where *N* is the number of the layers. *Z_k_*_+1_ and *Z_k_* are the distance from the top surface and bottom surface, respectively, to the middle surface of the *k*th layer. For analysis of the general cross-ply composite laminates shallow shell, the transform coefficients $\bar{Q_{16}}$, $\bar{Q_{26}}$ and $\bar{Q_{45}}$ are zero. So, the corresponding stiffness coefficients will be vanished

#### 2.2.2. Wave Function Solutions

The theoretical equations of the composite shell based on FSDT are given as follows [13]:

(8) $$\begin{array}{c} \frac{\partial N_{xx}}{\partial x}+\frac{\partial N_{xy}}{\partial y}+\frac{Q_{x}}{R_{x}}=I_{0}\frac{\partial^{2}u_{0}}{\partial t^{2}}+I_{1}\frac{\partial^{2}\phi_{x}}{\partial t^{2}}\\ \frac{\partial N_{yy}}{\partial y}+\frac{\partial N_{xy}}{\partial x}+\frac{Q_{y}}{R_{y}}=I_{0}\frac{\partial^{2}v_{0}}{\partial t^{2}}+I_{1}\frac{\partial^{2}\phi_{y}}{\partial t^{2}}\\ \frac{N_{xx}}{R_{x}}+\frac{N_{yy}}{R_{y}}-\frac{\partial Q_{x}}{\partial x}-\frac{\partial Q_{y}}{\partial y}=-I_{0}\frac{\partial^{2}w_{0}}{\partial t^{2}}\\ \frac{\partial M_{xx}}{\partial x}+\frac{\partial M_{xy}}{\partial y}-Q_{x}=I_{1}\frac{\partial^{2}u_{0}}{\partial t^{2}}+I_{2}\frac{\partial^{2}\phi_{x}}{\partial t^{2}}\\ \frac{\partial M_{yy}}{\partial y}+\frac{\partial M_{xy}}{\partial x}-Q_{y}=I_{1}\frac{\partial^{2}v_{0}}{\partial t^{2}}+I_{2}\frac{\partial^{2}\phi_{y}}{\partial t^{2}} \end{array}$$

where *I_i_* (*i* = 0, 1, 2) are the inertia mass moments. Submitting Equations (3) and (6) into Equation (8), the force vector and moment resultants can be transformed as displacement variables. Furthermore, the theoretical equations are follows:

(9) $$\left[\begin{array}{lllll} T_{11} & T_{12} & T_{13} & T_{14} & T_{15}\\ T_{21} & T_{22} & T_{23} & T_{24} & T_{25}\\ T_{31} & T_{32} & T_{33} & T_{34} & T_{35}\\ T_{41} & T_{42} & T_{43} & T_{44} & T_{45}\\ T_{51} & T_{52} & T_{53} & T_{54} & T_{55} \end{array}\right]\left\{\begin{array}{c} u_{0}\\ v_{0}\\ w_{0}\\ \phi_{x}\\ \phi_{y} \end{array}\right\}=\left\{\begin{array}{c} 0\\ 0\\ 0\\ 0\\ 0 \end{array}\right\}$$

where *T_ij_* (*i*,*j* = 1, 2, 3, 4, 5) are the operators of the matrix **T** in Equation (9), and are shown as follows:

(10) $$\begin{array}{l} T_{11}=A_{11\;}\frac{\partial^{2}}{\partial x^{2}}+A_{66}\;\frac{\partial^{2}}{\partial y^{2}}-\frac{K_{c}\;\;A_{55}}{R_{x}{}^{2}}-I_{0}\;\frac{\partial^{2}}{\partial t^{2}},T_{12}=\left(A_{66}+A_{12}\right)\frac{\partial^{2}}{\partial y\partial x},T_{13}=\left(\frac{A_{12}}{R_{y}}\;+\frac{A_{55}\;K_{c}+A_{1}{}_{1}}{R_{x}}\;\right)\frac{\partial }{\partial x}\\ T_{14}=B_{11\;}\frac{\partial^{2}}{\partial x^{2}}+B_{66}\;\;\frac{\partial^{2}}{\partial y^{2}}+\;\frac{K_{c}\;\;A_{5}5}{Rx}-I_{1\;}\frac{\partial^{2}}{\partial t^{2}},T_{15}=\left(B_{12}+B_{6}{}_{6}\right)\frac{\partial^{2}}{\partial y\partial x}\\ T_{21}=T_{12},T_{22}=A_{66}\;\frac{\partial^{2}}{\partial x^{2}}+A_{22}\;\frac{\partial^{2}}{\partial y^{2}}-\frac{K_{c}\;A_{4}4}{R_{y}{}^{2}}-I_{0\;}\frac{\partial^{2}}{\partial t^{2}},T_{23}=\left(\frac{A_{44}\;K_{c}+A_{22}}{\;R_{y}}+\frac{A_{12}}{R_{x}\;}\;\right)\frac{\partial }{\partial y}\\ T_{24}=\left(B_{12}+B_{66}\right)\frac{\partial^{2}}{\partial y\partial x},T_{25}=B_{66}\;\frac{\partial^{2}}{\partial x^{2}}+B_{2}{}_{2}\;\frac{\partial^{2}}{\partial y^{2}}+\;\frac{K_{c}\;A_{44}}{Ry}-I_{1}\;\frac{\partial^{2}}{\partial t^{2}}\\ T_{31}=T_{13},T_{23}=T_{32},T_{33}=-A_{55\;}K_{c}\frac{\partial^{2}}{\partial x^{2}}-A_{44}\;K_{c}\frac{\partial^{2}}{\partial y^{2}}+\left(\;\frac{A_{11}}{R_{x}{}^{2}}+\frac{2\;A_{1}{}_{2}\;}{R_{x}R_{y}}+\frac{A_{22}}{R_{y}{}^{2}}\;\right)+I_{0}\;\frac{\partial^{2}}{\partial t^{2}}\\ T_{34}=\left(-A_{55}\;K_{c}\;+\frac{B_{12}}{R_{y}}+\frac{B_{11}}{R_{x}}\right)\frac{\partial }{\partial x},T_{35}=-A_{44}\;K_{c}\;+\frac{B_{22}}{R_{y}}+\frac{B_{12}}{R_{x}}\;\\ T_{41}=T_{14},T_{42}=T_{24},T_{43}=T_{34},T_{44}=D_{11}\;\frac{\partial^{2}}{\partial x^{2}}+D_{66}\;\;\frac{\partial^{2}}{\partial y^{2}}-\;K_{c\;}A_{55}-I_{2}\;\frac{\partial^{2}}{\partial t^{2}}\\ T_{51}=T_{15},T_{52}=T_{25},T_{53}=T_{35},T_{54}=T_{45},T_{55}=D_{66}\;\frac{\partial^{2}}{\partial x^{2}}+D_{22}\;\frac{\partial^{2}}{\partial y^{2}}-K_{c}A_{44}-I_{2}\frac{\partial^{2}}{\partial t^{2}} \end{array}$$

For certain cross-ply composite laminated shallow shells under shear diaphragm boundary conditions, which are set at the opposite supports *y* = 0 and *Ly* (*u*_0_ = *w*_0_ = *ϕ_x_* = *N_yy_* = *M_yy_* = 0), the generalized displacement variables are transformed in the wave function form as follows:

(11) $$\left\{\begin{array}{c} u\left(x,y,t\right)\\ v\left(x,y,t\right)\\ w\left(x,y,t\right)\\ \phi_{x}\left(x,y,t\right)\\ \phi_{y}\left(x,y,t\right) \end{array}\right\}=\sum_{n=0}^{\infty }\left\{\begin{array}{c} U_{0}e^{ik_{n}x}\sin(K_{y}y)e^{-j\omega t}\\ V_{0}e^{ik_{n}x}\cos(K_{y}y)e^{-j\omega t}\\ W_{0}e^{ik_{n}x}\sin(K_{y}y)e^{-j\omega t}\\ \Phi_{x}e^{ik_{n}x}\sin(K_{y}y)e^{-j\omega t}\\ \Phi_{y}e^{ik_{n}x}\cos(K_{y}y)e^{-j\omega t} \end{array}\right\}$$

where *K_y_* = *n*π/*L_y_* is the *y* direction modal wave number and *k_n_* is the wave number in the x direction. *U*_0_, *V*_0_, *W*_0_, *Φ_x_*, and *Φ_y_* are the corresponding displacement amplitude variables of the *n*th mode for the composite laminated shallow shells.

Submitting the wave function solutions of the displacement variables into Equation (9), the governing equation can be obtained as follows:

(12) $$\left[\begin{array}{lllll} L_{11} & L_{12} & L_{13} & L_{14} & L_{15}\\ L_{21} & L_{22} & L_{23} & L_{24} & L_{25}\\ L_{31} & L_{32} & L_{33} & L_{34} & L_{35}\\ L_{41} & L_{42} & L_{43} & L_{44} & L_{45}\\ L_{51} & L_{52} & L_{53} & L_{54} & L_{55} \end{array}\right]\left\{\begin{array}{c} U_{0}\\ V_{0}\\ W_{0}\\ \Phi_{x}\\ \Phi_{x} \end{array}\right\}=\left\{\begin{array}{c} 0\\ 0\\ 0\\ 0\\ 0 \end{array}\right\}$$

where *L_ij_* (*i*,*j* = 1, 2, 3, 4, 5) are the governing equation coefficients of Equation (12), given as follows:

(13) $$\begin{array}{l} L_{11}=-k_{n}{}^{2}A_{11}-\;\frac{A_{55}\;K_{c}}{R_{x}{}^{2}}-K_{y}{}^{2}A_{66}+I_{0}\omega^{2},L_{12}=-ik_{n}K_{y}\left(A_{12}\;+A_{66}\right),L_{13}=ik_{n}\;\left(\frac{A_{11}}{R_{x}}+\frac{A_{12}}{R_{y}}+\frac{K_{c}A_{55}\;\;}{R_{x}}\right)\\ L_{14}=\frac{A_{55}\;K_{c}}{R_{x}}-k_{n}{}^{2}B_{11}-K_{y}{}^{2}B_{6}{}_{6}\;+I_{1}\omega^{2},L_{15}=-ik_{n}K_{y}\left(B_{12}+B_{66}\right)\\ L_{21}=-L_{12},L_{22}=-K_{y}{}^{2}A_{22}-\;\frac{K_{c}A_{44}\;}{R_{y}{}^{2}}-k_{n}{}^{2}A_{66}+I_{0}\;\omega^{2},L_{23}=K_{y}\left(\frac{A_{22}+A_{44}\;K_{c}\;}{\;R_{y}}+\frac{A_{12}}{R_{x}\;}\right)\\ L_{24}=ik_{n}K_{y}\left(B_{12}+B_{66}\;\right),L_{25}=-K_{y}{}^{2}B_{22}-k_{n}{}^{2}B_{66}+\;\frac{K_{c}A_{44}\;}{\;R_{y}}\;+I_{1}\;\omega^{2}\\ L_{31}=L_{13},L_{32}=-L_{23},L_{33}=\frac{A_{11}\;}{R_{x}{}^{2}}+\;\frac{2\;A_{12}}{R_{x}R_{y}}+\frac{A_{22}}{R_{y}{}^{2}}+K_{c}\;k_{n}{}^{2}A_{55}+K_{c}K_{y}{}^{2}A_{44}-I_{0\;}\omega^{2}\\ L_{34}=i\;k_{n}\left(\frac{B_{12}\;}{R_{y}}+\frac{B_{11}\;}{R_{x}}-K_{c\;}A_{55}\;\right),L_{35}=K_{y}\left(K_{c}\;A_{44}-\frac{\;B_{12}\;}{R_{x}}-\frac{B_{22}\;}{R_{y}}\right)\\ L_{41}=-L_{14},L_{42}=L_{24},L_{43}=-L_{34},L_{44}=k_{n}{}^{2}D_{11}+K_{y}{}^{2}D_{66}+K_{c}A_{55}-I_{2}\omega^{2}\\ L_{51}=L_{15},L_{52}=-L_{25},L_{53}=L_{35},L_{54}=-L_{45},L_{55}=K_{y}{}^{2}D_{22}+\;k_{n}{}^{2}D_{66}+K_{c}A_{44}-I_{2}\;\omega^{2} \end{array}$$

The solutions of Equation (12) can be solved and the determinant of the matrix **T** equal to zero. The characteristics equation of axial wavenumber *k_n_* is shown as follows:

(14) $$\lambda_{10}k_{n}^{10}+\lambda_{8}k_{n}^{8}+\lambda_{6}k_{n}^{6}+\lambda_{4}k_{n}^{4}+\lambda_{2}k_{n}^{2}+\lambda_{0}=0$$

There is a fifth-order equation of $k_{n}^{2}$ and *λ*_10_, *λ*_8_, *λ*_6_, *λ*_4_, *λ*_2_, and *λ*_0_ are the coefficients, which depend on the coefficient matrix **T**. There are ten characteristic axial wavenumbers to be obtained as ±*k_n_*_,1_, ±*k_n_*_,2_, ±*k_n_*_,3_, ±*k_n_*_,4_, and ±*k_n_*_,5_. Through the characteristic axial wavenumbers ±*k_n_*_,*i*_ (*i* = 1, 2, 3, 4, 5), the corresponding basic solution vector is defined as follows:

(15) $$\left\{\xi_{n,i},\eta_{n,i},1,\chi_{n,i},\psi_{n,i}\right\}$$

The coefficients in Equation (15) are defined as follows:

(16) $$\xi_{n,i}={\left[\frac{\Omega_{1}}{\Omega }\right]}_{k_{n}=\pm k_{n,i}},\eta_{n,i}={\left[\frac{\Omega_{2}}{\Omega }\right]}_{k_{n}=\pm k_{n,i}},\chi_{n,i}={\left[\frac{\Omega_{4}}{\Omega }\right]}_{k_{n}=\pm k_{n,i}},\psi_{n,i}={\left[\frac{\Omega_{5}}{\Omega }\right]}_{k_{n}=\pm k_{n,i}}$$

where Ω and Ω*_i_* (*i* = 1, 2, 4, 5) are given as follows:

(17) $$\begin{array}{lll} \Omega ={\left|\begin{array}{llll} L_{11} & L_{12} & L_{14} & L_{15}\\ L_{21} & L_{22} & L_{24} & L_{25}\\ L_{41} & L_{42} & L_{44} & L_{45}\\ L_{51} & L_{52} & L_{54} & L_{55} \end{array}\right|}_{k_{n}=\pm k_{n,i}} & \Omega_{1}={\left|\begin{array}{llll} -L_{13} & L_{12} & L_{14} & L_{15}\\ -L_{23} & L_{22} & L_{24} & L_{25}\\ -L_{43} & L_{42} & L_{44} & L_{45}\\ -L_{53} & L_{52} & L_{54} & L_{55} \end{array}\right|}_{k_{n}=\pm k_{n,i}} & \Omega_{2}={\left|\begin{array}{llll} L_{11} & -L_{13} & L_{14} & L_{15}\\ L_{21} & -L_{23} & L_{24} & L_{25}\\ L_{41} & -L_{43} & L_{44} & L_{45}\\ L_{51} & -L_{53} & L_{54} & L_{55} \end{array}\right|}_{k_{n}=\pm k_{n,i}}\\ \Omega_{4}={\left|\begin{array}{llll} L_{11} & L_{12} & -L_{13} & L_{15}\\ L_{21} & L_{22} & -L_{23} & L_{25}\\ L_{41} & L_{42} & -L_{43} & L_{45}\\ L_{51} & L_{52} & -L_{53} & L_{55} \end{array}\right|}_{k_{n}=\pm k_{n,i}} & \Omega_{5}={\left|\begin{array}{llll} L_{11} & L_{12} & L_{14} & -L_{13}\\ L_{21} & L_{22} & L_{24} & -L_{23}\\ L_{41} & L_{42} & L_{44} & -L_{43}\\ L_{51} & L_{52} & L_{54} & -L_{53} \end{array}\right|}_{k_{n}=\pm k_{n,i}} & \end{array}$$

On the basis of the generalized displacement variables being transformed in the wave function form in Equation (11), related to the basic solution vector in Equation (15), the generalized displacement variables are shown in the matrix form:

(18) $$\delta_{n}=Y_{n}\left(y\right)D_{n}P_{n}\left(x\right)W_{n}$$

where **δ***_n_* = {*u*, *v*, *w*, *ϕ_x_*, *ϕ_y_*}*^T^* is the generalized displacement resultant; **Y***_n_*(*y*) is the modal matrix in the y direction; **D***_n_* is the coefficient matrix of the displacement resultant; **P***_n_*(*x*) is the axial wavenumber matrix; and **W***_n_* is the wave contribution factor resultant. The detailed expression of them is given as follows:

(19) $$Y_{n}\left(y\right)=diag\left\{\sin\left(K_{y}y\right),\cos\left(K_{y}y\right),\sin\left(K_{y}y\right),\sin\left(K_{y}y\right),\cos\left(K_{y}y\right)\right\}$$

(20) $$D_{n}=\left[\begin{array}{ccccc} \xi_{n,1} & \xi_{n,2} & \cdots & \xi_{n,ns-1} & \xi_{n,ns}\\ \eta_{n,i} & \eta_{n,i} & \cdots & \eta_{n,ns-1} & \eta_{n,ns}\\ 1 & 1 & \cdots & 1 & 1\\ \chi_{n,1} & \chi_{n,2} & \cdots & \chi_{n,ns} & \chi_{n,ns-1}\\ \psi_{n,1} & \psi_{n,2} & \cdots & \psi_{n,ns} & \psi_{n,ns-1} \end{array}\right]$$

(21) $$P_{n}\left(x\right)=diag\left\{e^{ik_{n,1}x},e^{ik_{n,2}x},\cdots ,e^{ik_{n,ns-1}x},e^{ik_{n,ns}x}\right\}$$

(22) $$W_{n}={\left\{W_{n,1},W_{n,2},\cdots ,W_{n,ns-1},W_{n,ns}\right\}}^{T}$$

where *ns* is the number of characteristics roots of axial wavenumber in Equation (14). Also, the generalized force resultant **f***_n_* = {*N_xx_*, *N_xy_*, *Q_x_*, *M_xx_*, *M_xy_*}*^T^* can refer to the constitutive relationship in Equations (3) and (6), as follows:

(23) $$f_{n}=Y_{n}\left(y\right)F_{n}P_{n}\left(x\right)W_{n}$$

where the coefficient matrix **F***_n_* of force resultant **f***_n_* is given as follows:

(24) $$\begin{array}{l} F_{n,1i}=ik_{n,i}A_{11}\;\xi_{n,i}-K_{y}A_{12}\eta_{n,i}+\frac{A_{11}\;}{R_{x}}+\frac{A_{12\;}}{\;R_{y}}+ik_{n,i}B_{11}\;\chi_{n,i}\;-K_{y}B_{12}\;\psi_{n,i}\\ F_{n,2i}=K_{y}A_{66}\xi_{n,i}+ik_{n,i}A_{66}\;\eta_{n,i}+K_{y}B_{66}\chi_{n,i}+i\;k_{n,i}B_{66}\;\psi_{n,i}\\ F_{n,3i}=K_{c}\;A_{55}\left(ik_{n,i}+\;\chi_{n,i}-\frac{\xi_{n,i}}{R_{x}}\right)\\ F_{n,4i}=ik_{n,i}B_{11}\;\;\xi_{n,i}-K_{y}B_{12}\;\eta_{n.i}\;+\frac{B_{11\;}}{R_{x}\;}+\frac{B_{12}\;}{R_{y}}+ik_{n,i}D_{11}\;\chi_{n,i}\;-K_{y}D_{12\;}\psi_{n,i}\\ F_{n,5i}=K_{y}B_{66}\;\xi_{n,i}+ik_{n,i}B66\eta_{n,i}+K_{y}D_{66}\;\chi_{n,i}+i\;k_{n,i}D_{66}\;\psi_{n,i}\; \end{array}$$

### 2.3. Classical Shell Theory (CST)

#### 2.3.1. Kinematic Relations and Stress Resultants

For the integrity of the paper, the governing equations and wave function solutions in the CST are given. On the basis of the theoretical technique of FSDT, the governing equation can refer to CST by setting the slope of the rotation components *ϕ_x_* and *ϕ_y_* close to the transverse normal, as follows [12,13]:

(25) $$\phi_{x}=\frac{u_{0}}{R_{x}}-\frac{\partial w_{0}}{\partial x},\phi_{y}=\frac{v_{0}}{R_{y}}-\frac{\partial w_{0}}{\partial y}$$

#### 2.3.2. Wave Function Solutions

In CST, the shear deformation in the kinematics equation is negligible, and the in-plane displacement can be expressed as a linear change in the thickness direction of the shallow shell. So, the governing equation can be given as follows [13]:

(26) $$\begin{array}{c} \frac{\partial N_{xx}}{\partial x}+\frac{\partial N_{xy}}{\partial y}+\frac{Q_{x}}{R_{x}}=I_{0}\frac{\partial^{2}u_{0}}{\partial t^{2}}\\ \frac{\partial N_{yy}}{\partial y}+\frac{\partial N_{xy}}{\partial x}+\frac{Q_{y}}{R_{y}}=I_{0}\frac{\partial^{2}v_{0}}{\partial t^{2}}\\ \frac{N_{xx}}{R_{x}}+\frac{N_{yy}}{R_{y}}-\left(\frac{\partial Q_{x}}{\partial x}+\frac{\partial Q_{y}}{\partial y}\right)=-I_{0}\frac{\partial^{2}w_{0}}{\partial t^{2}} \end{array}$$

where

(27) $$\begin{array}{c} Q_{x}=\frac{\partial M_{xx}}{\partial x}+\frac{\partial M_{xy}}{\partial y}\\ Q_{y}=\frac{\partial M_{yy}}{\partial y}+\frac{\partial M_{xy}}{\partial x} \end{array}$$

Submitting the generalized displacement variables in CST, the governing equation can be expressed as follows:

(28) $$\left[\begin{array}{ccc} {\tilde{T}}_{11} & {\tilde{T}}_{12} & {\tilde{T}}_{13}\\ {\tilde{T}}_{21} & {\tilde{T}}_{22} & {\tilde{T}}_{23}\\ {\tilde{T}}_{31} & {\tilde{T}}_{32} & {\tilde{T}}_{33} \end{array}\right]\left\{\begin{array}{c} u_{0}\\ v_{0}\\ w_{0} \end{array}\right\}=\left\{\begin{array}{c} 0\\ 0\\ 0 \end{array}\right\}$$

where ${\tilde{T}}_{ij}$ (*i*,*j* = 1, 2, 3) are the operators, which are shown as follows:

(29) $$\begin{array}{l} {\tilde{T}}_{11}=\left(A_{11}+\frac{2B_{11}}{R_{x}}+\frac{D_{11}}{R_{x}{}^{2}}\right)\frac{\partial^{2}}{\partial x^{2}}+\left(A_{6}{}_{6}+\frac{2B_{66}\;\;}{R_{x}}+\frac{\;D_{66}}{R_{x}{}^{2}}\right)\;\frac{\partial^{2}}{\partial y^{2}}-I_{0}\;\frac{\partial^{2}}{\partial t^{2}}\\ {\tilde{T}}_{12}=\left(A_{12}+A_{66}+\frac{B_{12}+B_{66}}{R_{y}}+\frac{B_{12}+B_{66}}{R_{x}}+\frac{D_{12}+D_{66}}{R_{x}R_{y}}\right)\frac{\partial^{2}}{\partial y\partial x}\\ {\tilde{T}}_{13}=-\left(B_{11}+\frac{D_{11}}{R_{x}}\right)\frac{\partial^{3}}{\partial x^{3}}+\left(\frac{A_{12}\;}{R_{y}}+\;\frac{A_{11}}{R_{x}}+\frac{B_{12}}{R_{x}R_{y}}+\frac{B_{1}{}_{1}}{R_{x}{}^{2}}\;\right)\frac{\partial }{\partial x}-\;\left(B_{12}+2\;B_{66}+\frac{D_{12}+2\;D_{66}}{R_{x}}\right)\frac{\partial^{3}}{\partial y^{2}\partial x}\\ {\tilde{T}}_{21}={\tilde{T}}_{12},{\tilde{T}}_{22}=\left(A_{66}+\frac{2B_{66}\;}{\;R_{y}}\;+\;\frac{D_{66}}{\;R_{y}{}^{2}}\right)\frac{\partial^{2}}{\partial x^{2}}+\;\left(A_{22}+\frac{2B_{22}\;}{\;R_{y}}+\frac{D_{22}}{\;R_{y}{}^{2}}\;\right)\;\frac{\partial^{2}}{\partial y^{2}}-I_{0}\;\frac{\partial^{2}}{\partial t^{2}}\\ {\tilde{T}}_{23}=-\;\left(B_{22\;}+\frac{D_{22}}{R_{y}}\right)\frac{\partial^{3}}{\partial y^{3}}-\;\;\left(2B_{66}+\;B_{12}+\frac{D_{12}+2D_{66}}{R_{y}}\right)\frac{\partial^{3}}{\partial y\partial x^{2}}+\left(\;\frac{A_{12}}{R_{x}\;}+\;\frac{A_{22}}{R_{y}}+\frac{B_{12}}{R_{x}\;R_{y}}+\frac{B_{22}}{\;R_{y}{}^{2}}\;\right)\frac{\partial }{\partial y}\\ {\tilde{T}}_{31}={\tilde{T}}_{13},{\tilde{T}}_{32}={\tilde{T}}_{23}\\ \begin{array}{l} {\tilde{T}}_{33}=D_{11}\;\frac{\partial^{4}}{\partial x^{4}}+D_{22}\frac{\partial^{4}}{\partial y^{4}}+2\left(D_{12}+2\;D_{66}\right)\frac{\partial^{4}}{\partial y^{2}\partial x^{2}}+\left(-\;\frac{2\;B_{11}}{R_{x}}-\frac{2\;B_{12}\;}{R_{y}}\right)\frac{\partial^{2}}{\partial x^{2}}\\ +\left(-\;\frac{2\;B_{12}}{R_{x}}-\frac{2\;B_{22}}{R_{y}}\;\right)\frac{\partial^{2}}{\partial y^{2}}+\left(\frac{A_{11}}{R_{x}{}^{2}}+\frac{2\;A_{12}}{R_{x\;}R_{y}}\;+\frac{A_{22}}{R_{y}{}^{2}}\;\right)+I_{0}\;\frac{\partial^{2}}{\partial t^{2}} \end{array} \end{array}$$

For the generalized displacement functions of cross-ply composite laminated shallow shell with shear diaphragm boundary conditions, which are set as opposite support edges *y* = 0 and *Ly* (*u*_0_ = *w*_0_ = *N_yy_* = *M_yy_* = 0), the displacement variables can be shown in the wave functions form:

(30) $$\left\{\begin{array}{c} u\left(x,y,t\right)\\ v\left(x,y,t\right)\\ w\left(x,y,t\right)\\ \phi_{x}\left(x,y,t\right) \end{array}\right\}=\sum_{n=0}^{\infty }\left\{\begin{array}{c} U_{0}e^{ik_{n}x}\sin\left(K_{y}y\right)e^{-j\omega t}\\ V_{0}e^{ik_{n}x}\cos\left(K_{y}y\right)e^{-j\omega t}\\ W_{0}e^{ik_{n}x}\sin\left(K_{y}y\right)e^{-j\omega t}\\ \Phi_{x}e^{ik_{n}x}\sin\left(K_{y}y\right)e^{-j\omega t} \end{array}\right\}$$

Submitting Equation (30) into Equation (28), the governing equation can transform into the matrix form as follows:

(31) $$\left[\begin{array}{ccc} {\tilde{L}}_{11} & {\tilde{L}}_{12} & {\tilde{L}}_{13}\\ {\tilde{L}}_{21} & {\tilde{L}}_{22} & {\tilde{L}}_{23}\\ {\tilde{L}}_{31} & {\tilde{L}}_{32} & {\tilde{L}}_{33} \end{array}\right]\left\{\begin{array}{c} U_{0}\\ V_{0}\\ W_{0} \end{array}\right\}=\left\{\begin{array}{c} 0\\ 0\\ 0 \end{array}\right\}$$

where ${\tilde{L}}_{ij}$ (*i*,*j* = 1, 2, 3) are the governing equation coefficients, as follows:

(32) $$\begin{array}{l} {\tilde{L}}_{11}=-\;k_{n}{}^{2}\left(A_{11}+\;\frac{2\;B_{11}}{R_{x}}+\;\frac{D_{11}}{R_{x}{}^{2}}\right)-K_{y}{}^{2}\left(A_{66}+\;\frac{2\;B_{66}}{R_{x}}+\frac{D_{66}}{R_{x}{}^{2}}\right)+I_{0}\omega^{2}\\ {\tilde{L}}_{12}=-ik_{n}K_{y}\left(A_{12}+A_{66}\;+\frac{B_{66}+B_{12}\;}{R_{y}}\;+\frac{B_{66}+B_{12}}{R_{x}}+\frac{D_{12}+D_{66}}{R_{x}R_{y}}\;\right)\\ {\tilde{L}}_{13}=ik_{n}{}^{3}\left(B_{11}+\frac{D_{11}\;}{R_{x}}\right)+ik_{n}\left(\frac{A_{11}\;}{R_{x}}+\;\frac{A_{12}}{R_{y}}+\frac{B_{11}}{R_{x}{}^{2}}+\frac{B_{12}}{R_{x}R_{y}}\right)+ik_{n}K_{y}{}^{2}\left(B_{12}+2B_{66}+\;\frac{D_{12}+2\;D_{66}}{R_{x}}\right)\\ {\tilde{L}}_{21}=-{\tilde{L}}_{12},{\tilde{L}}_{22}=-k_{n}{}^{2}\left(A_{66}+\frac{2\;B_{66}}{R_{y}}+\;\frac{D_{66}}{R_{y}{}^{2}}\;\right)\;\;-K_{y}{}^{2}\left(A_{22}+\frac{2\;B_{22}}{R_{y}}+\frac{D_{22}\;}{R_{y}{}^{2}}\right)+I_{0\;}\omega^{2}\;\\ {\tilde{L}}_{23}=\;K_{y}{}^{3}\left(B_{22}+\frac{D_{22}}{Ry}\right)\;+k_{n}{}^{2}K_{y}\left(2\;B_{66}+B_{12\;}+\;\frac{D_{12}+2\;D_{66}}{\;R_{y}}\;\right)+K_{y}\left(\frac{A_{12}}{R_{x\;}}+\frac{A_{22}\;}{\;R_{y}}+\;\frac{B_{12}}{R_{x\;}R_{y}}+\;\frac{B_{22}}{R_{y}{}^{2}}\;\right)\\ \begin{array}{l} {\tilde{L}}_{31}={\tilde{L}}_{13},{\tilde{L}}_{32}=-{\tilde{L}}_{23},{\tilde{L}}_{33}=k_{n}{}^{4}D_{11}+K_{y}{}^{4}D_{22}+2\;k_{n}{}^{2}K_{y}{}^{2}\left(D_{12}+2D_{66}\right)+2k_{n}{}^{2}\left(\frac{\;B_{11}}{R_{x}}+\;\frac{B_{12}}{R_{y}}\right)\\ +2\;K_{y}{}^{2}\left(\frac{B_{12}}{R_{x}}+\;\frac{\;B_{22}}{R_{y}}\right)+\left(\frac{A_{11}}{R_{x}{}^{2}}+\;\frac{A_{22}}{R_{y}{}^{2}}+\;\frac{2\;A_{12}}{R_{x}R_{y}}\right)\;-I_{0}\omega^{2}\; \end{array} \end{array}$$

Next, the corresponding basic solution vector is set as {*ξ_n,i_, η_n,i_*, 1}*^T^*, and the detailed expression of the vector is given as follows:

(33) $$\xi_{n,i}={\left[\frac{\Omega_{1}}{\Omega }\right]}_{k_{n}=\pm k_{n,i}},\eta_{n,i}={\left[\frac{\Omega_{2}}{\Omega }\right]}_{k_{n}=\pm k_{n,i}}$$

where

(34) $$\Omega ={\left|\begin{array}{cc} N_{11} & N_{12}\\ N_{21} & N_{22} \end{array}\right|}_{k_{n}=\pm k_{n,i}},\Omega_{1}={\left|\begin{array}{cc} -N_{13} & N_{12}\\ -N_{23} & N_{22} \end{array}\right|}_{k_{n}=\pm k_{n,i}},\Omega_{2}={\left|\begin{array}{cc} N_{11} & -N_{13}\\ N_{21} & -N_{23} \end{array}\right|}_{k_{n}=\pm k_{n,i}}$$

On the basis of the basic solution vector, the displacement resultant **δ**_n_ = {*u*, *v*, *w*, *ϕx*}*^T^* and force resultant **f***_n_* = {*N_xx_*,*N_xy_* + *M_xy_*/*R_y_*,*Q_x_* + ∂*M_xy_*_/_∂*_y_*,*M_xx_*}*^T^* are expressed as follows:

(35) $$\begin{array}{l} \delta_{n}=Y_{n}\left(y\right)D_{n}P_{n}\left(x\right)W_{n}\\ f_{n}=Y_{n}\left(y\right)F_{n}P_{n}\left(x\right)W_{n} \end{array}$$

where

(36) $$Y_{n}\left(y\right)=diag\left\{\sin\left(K_{y}y\right),\cos\left(K_{y}y\right),\sin\left(K_{y}y\right),\sin\left(K_{y}y\right)\right\}$$

(37) $$D_{n}=\left[\begin{array}{ccccc} \xi_{n,1} & \xi_{n,2} & \cdots & \xi_{n,ns-1} & \xi_{n,ns}\\ \eta_{n,1} & \eta_{n,2} & \cdots & \eta_{n,ns-1} & \eta_{n,ns}\\ 1 & 1 & \cdots & 1 & 1\\ \frac{\xi_{n,1}}{R_{x}}-i\;k_{n,1} & \frac{\xi_{n,2}}{R_{x}}-i\;k_{n,2} & \cdots & \frac{\xi_{n,ns-1}}{R_{x}}-i\;k_{n,ns-1} & \frac{\xi_{n,ns}}{R_{x}}-i\;k_{n,ns} \end{array}\right]$$

(38) $$F_{n}=\left[\begin{array}{ccccc} F_{n,11} & F_{n,12} & \cdots & F_{n,1ns-1} & F_{n,1ns}\\ F_{n,21} & F_{n,22} & \cdots & F_{n,2ns-1} & F_{n,2ns}\\ F_{n,31} & F_{n,32} & \cdots & F_{n,3ns-1} & F_{n,3ns}\\ F_{n,41} & F_{n,42} & \cdots & F_{n,4ns-1} & F_{n,4ns} \end{array}\right]$$

in which the coefficients *F_n_*_,*ji*_(*j* = 1–4,*i* = 1–*ns*) are given as follows:

(39) $$\begin{array}{l} F_{n,1i}=ik_{n,i}\left(A_{11}+\;\frac{B_{11}}{R_{x}\;}\;\right)\;\xi_{n,i}+K_{y}\left(-A_{12}-\frac{B_{12}}{R_{y}}\right)\eta_{n,i}+\frac{A_{11}}{R_{x}\;}+\frac{A_{12}}{\;R_{y}}+k_{n,i}{}^{2}B_{11}+K_{y}{}^{2}B_{12}\\ F_{n,2i}=K_{y}\left(A_{66}+\;\frac{B_{66}}{R_{x}\;}+\;\frac{B_{66}}{\;R_{y}}+\frac{D_{66}}{R_{x}\;R_{y}}\right)\;\xi_{n,i}\;+ik_{n,i}\left(A_{66}+\frac{2B_{66}}{R_{y}}+\frac{D_{66}}{R_{y}{}^{2}}\right)\;\eta_{n,i}+2ik_{n,i}K_{y}\left(-B_{66}-\frac{D_{66}}{R_{y}}\right)\;\\ \begin{array}{l} F_{n,3i}=-\left(k_{n,i}{}^{2}\left(B_{11}+\frac{D_{11}}{R_{x}\;}\;\right)\;+2K_{y}{}^{2}\left(B_{66}+\;\frac{D_{66}}{R_{x}\;}\right)\right)\xi_{n,i}\;-ik_{n,i}K_{y}\left(B_{12}+2\;B_{66}+\frac{D_{12}+2\;D_{66}\;}{R_{y}}\right)\;\eta_{n,i}\\ +ik_{n,i}{}^{3}D_{11}+ik_{n,i}K_{y}{}^{2}\left(D_{12}+4D_{66}\right)+ik_{n,i}\left(\frac{B_{11}}{R_{x}}+\frac{B_{12}}{R_{y}}\right)\; \end{array}\\ F_{n,4i}=ik_{n,i}\left(B_{11}\;+\;\frac{D_{11}}{R_{x}\;}\;\right)\xi_{n,i}-K_{y}\left(B_{12}+\frac{D_{12}\;}{R_{y}}\right)\eta_{n.i}\;+\;k_{n,i}{}^{2}D_{11}\;\;+K_{y}{}^{2}D_{12}+\frac{B_{11}}{R_{x}\;}+\;\frac{B_{12}}{\;R_{y}} \end{array}$$

### 2.4. Implementation of the WBM

Through the introduction of the generalized displacement and force resultant, the final governing equations are assembled by the generalized displacement coefficient matrix, generalized force coefficient matrix, and boundary matrix. The final governing equation of the whole structure is defined as follows:

(40) [K]{W}={F}

where **F** is the external force vector and is related to the external situation; when analyzing the free vibration dynamic, the external force **F** should vanish. **W** = {**W**_1_, **W**_2_}*^T^* is the wave contribution factor resultant of the composite shell, and **W***_i_* = {*W_i_*_,1_, *W_i_*_,2_,…, *W_i_*_,*ns*_}*^T^*(*i* = 1, 2) is the wave contribution factor vector and is associated with the boundary conditions at *x* = 0 and *x* = *L*. **K** is the total matrix and the detailed expression of the matrix is shown as follows:

(41) K2ns×2ns=[B1(0)012ns×nsDnPn(L)−DnPn(0)FnPn(L)−FnPn(0)012ns×nsB2(0)]

For the classical boundary conditions, the boundary matrix can be shown as follows:

(42) $$B_{1,2}\left(x\right)=\left(T_{\delta }D_{n}+T_{f}F_{n}\right)P_{n}\left(x\right)$$

where **T***_δ_* and **T***_f_* are the transform matrix of boundary matrix, as follows:

Free edge (*F*):

(43) $$\begin{array}{c} \mathrm{FSDT}:\left\{ \begin{array}{l} T_{\delta }=diag\left\{0,0,0,0,0\right\}\\ T_{f}=diag\left\{1,1,1,1,1\right\} \end{array}\right.\\ \mathrm{CST}:\left\{ \begin{array}{l} T_{\delta }=diag\left\{0,0,0,0\right\}\\ T_{f}=diag\left\{1,1,1,1\right\} \end{array}\right. \end{array}$$

Clamped edge (*C*):

(44) $$\begin{array}{c} \mathrm{FSDT}:\left\{ \begin{array}{l} T_{\delta }=diag\left\{1,1,1,1,1\right\}\\ T_{f}=diag\left\{0,0,0,0,0\right\} \end{array}\right.\\ \mathrm{CST}:\left\{ \begin{array}{l} T_{\delta }=diag\left\{1,1,1,1\right\}\\ T_{f}=diag\left\{0,0,0,0\right\} \end{array}\right. \end{array}$$

Shear-diaphragm edge (*SD*):

(45) $$\begin{array}{c} \mathrm{FSDT}:\left\{ \begin{array}{l} T_{\delta }=diag\left\{0,1,1,0,1\right\}\\ T_{f}=diag\left\{1,0,0,1,0\right\} \end{array}\right.\\ \mathrm{CST}:\left\{ \begin{array}{l} T_{\delta }=diag\left\{0,1,1,0\right\}\\ T_{f}=diag\left\{1,0,0,1\right\} \end{array}\right. \end{array}.$$

For the elastic boundary conditions, the boundary condition matrix **B**_1_(*x*) and **B**_2_(*x*) are given as follows:

(46) $$B_{1,2}\left(x\right)=\left(K_{\delta }D_{n}\pm F_{n}\right)P_{n}\left(x\right),$$

where **K***_δ_* is the stiffness transform matrix and the detailed expression about it is as follows:

When the composite shell is under elastic restraint in the axial direction, the stiffness transform matrix **K***_δ_* is given as follows:

(47) $$\begin{array}{c} \mathrm{FSDT}:\\ \mathrm{CST}: \end{array}\{\begin{array}{c} K_{\delta }=diag\left\{K_{u},0,0,0,0\right\}\\ K_{\delta }=diag\left\{K_{u},0,0,0\right\} \end{array}$$

where {*K_u_*, *K_v_*, *K_w_* } are linear springs and {*K_ϕx_*, *K_ϕy_* } are rotational springs, which are set in various directions. When the other displacements are under elastic restraint, the stiffness transform matrix **K***_δ_* is given as follows:

(48) $$\begin{array}{ll} v: & \begin{array}{r} \mathrm{FSDT}:\\ \mathrm{CST}: \end{array}\{\begin{array}{l} K_{\delta }=diag\left\{0,K_{v},0,0,0\right\}\\ K_{\delta }=diag\left\{0,K_{v},0,0\right\} \end{array}\\ w: & \begin{array}{r} \mathrm{FSDT}:\\ \mathrm{CST}: \end{array}\{\begin{array}{l} K_{\delta }=diag\left\{0,0,K_{w},0,0\right\}\\ K_{\delta }=diag\left\{0,0,K_{w},0\right\} \end{array}\\ \phi_{x}: & \begin{array}{r} \mathrm{FSDT}:\\ \mathrm{CST}: \end{array}\{\begin{array}{l} K_{\delta }=diag\left\{0,0,0,K_{\phi x},0\right\}\\ K_{\delta }=diag\left\{0,0,0,K_{\phi x}\right\} \end{array}\\ \phi_{y}: & \mathrm{FSDT}:K_{\delta }=diag\left\{0,0,0,0,K_{\phi y}\right\} \end{array}$$

Through the introduction of the boundary conditions **B**_1_(*x*) and **B**_2_(*x*), which include the classical and elastic boundary conditions, the total matrix **K** is established. When analyzing the free vibration characteristics, the external force vector F vanishes. When calculating the natural frequencies, a series of the total matrix determinant is obtained. Using the dichotomy method to search the zeros position of the total matrix determinant, the natural frequency will be obtained with each circumferential mode number *n*. Through the numerical dichotomy method when the sign changed, the location of the total matrix **K** determinant is calculated and the natural frequencies can be obtained. Furthermore, to analyze the free vibration characteristics of the composite laminated shallow shell with arbitrary boundary conditions, the shell structure is considered to be calculated as a whole model and the displacement variable solutions are set as infinite wave function forms; the convergence study of the truncated number does not need to be considered. Thus, the computational cost of the present approach is low.

## 3. Numerical Examples and Discussion

Through the description of the theory formulation with FSDT and CST, the free vibration characteristics of composite laminated shallow shell with arbitrary classical boundary conditions, elastic boundary conditions, and their combinations are analyzed by WBM. In this part, some numerical examples are listed to verify the correctness of the results by WBM through the comparison with the presented results. Also, some numerical examples are presented to study the influence of the material parameters and geometric constants on the natural frequencies of composite laminated shallow shells with general boundary conditions.

### 3.1. Composite Laminated Shallow Shell with Classical Boundary Conditions

In this section, the free vibration characteristics of composite laminated shallow shells with arbitrary classical boundary conditions are concerned. Through the introduction of the boundary transform matrix **T***_δ_* and **T***_f_*, arbitrary classical boundary conditions can transform into boundary matrices **B**_1_(*x*) and **B**_2_(*x*) to investigate the free vibration characteristics of composite shallow shell with classical boundary conditions. In order to verify the correctness of the calculation by the presented method, some numerical examples are selected for verification. At the same time, the selected material parameters and geometric parameters are consistent with the examples in the comparative literatures. 

First, the composite laminated shallow shell with full shear diagram boundary condition is concerned. In Table 1 and Table 2, the fundamental frequency parameters $\Omega =\omega L_{x}^{2}\sqrt{\rho /E_{2}h^{2}}$ for three type cross-ply composite laminated shallow shells (i.e., cylindrical shell, spherical shell, and hyperbolic paraboloidal shell) with various radius to length ratios *R_y_*/*L_y_* (i.e., *R_y_*/*L_y_* = 2, 5, 10) under Shear-diaphragm boundary condition (SD-SD) by FSDST and CST are presented. Three kinds of cross-ply type layered composite shells (i.e., [0°/90°/90°/0°], [0°/90°], and [90°/0°]) are concerned. The material parameters and geometric constants are given as follows: *L_x_* = 1 m, *L_y_*/*L_x_* = 1, *h*/*L_y_* = 0.01 and 0.1, *E*_2_ = 7 GPa, *E*_1_/*E*_2_ = 15, *G*_12_ = *G*_13_ = 0.5*E*_2_, *G*_23_ = 0.5*E*_2_, *μ*_12_ = 0.25, *ρ* = 1650 kg/m^3^. The presented results compare with the results by Qatu [13] and Shao et al. [22]. From Table 1 and Table 2, the presented results by WBM match well with the results in the presented literatures. The maximum divergence is −4.61% with the situation **R_y_/L_y_** = −1 for the [90°/0°] cross-ply composite laminated paraboloidal shell. It is obvious that the errors in Table 2 by CST are lower than the errors in Table 1 by FSDT. Also, from Table 1, it can be found that, with the radius to length ratios *R_y_*/*L_y_* from 2 to 10 for the composite shallow shell with the lamination schemes 0°/90°/90°/0° and 0°/90°, the errors between the solutions by the presented method those of the the results in the literature by Quta are generally growing. This is caused by the curvature effect, which is not well predicted by shallow shell theory, thus full shell theory should be considered. Furthermore, when the parameter (*R_x_*/*R_y_*) decreases from 1 to −1, the fundamental frequency parameters for the composite laminates are lower. It can be observed that the fundamental frequency parameter Ω for the composite laminated spherical shell is higher than that for the cylindrical shell and hyperbolic paraboloidal shell.

In the next part, the fundamental frequency parameters Ω of a composite laminated plate with SD-SD boundary conditions are compared with the results by Qatu [13] in Table 3 and Table 4. In Table 3, two types of layered cross-ply composite laminated plates (i.e., [0°/90°] and [0°/90°/90°/0°]) by FSDT and CST are investigated with various length to thickness ratios *L_y_*/*h* (i.e., *L_y_*/*h* = 5, 10, 20, and 100). The material constants and geometric parameters are set as follows: *L_x_* = 1 m, *L_y_*/*L_x_* = 1, *E*_2_ = 7 GPa, *E*_1_/*E*_2_ = 15, *G*_12_ = *G*_13_ = 0.5*E*_2_, *G*_23_ = 0.5*E*_2_, *μ*_12_ = 0.25, *ρ* = 1650 kg/m^3^. From Table 3, it is clearly seen that the results by the presented method agree well with the solutions in the presented literatures. Also, with the growing of the length to thickness ratios *L_y_*/*h*, the fundamental frequency parameters are decreased for the two types of layered cross-ply composite laminated plates by different theory. Particularly, the fundamental frequency parameter is basically unchanged with [0°/90°/90°/0°] cross-ply composite laminated plate by CST. Furthermore, three types of layered cross-ply composite laminated plates (i.e., [0°/90°], [0°/90°/0°], and [0°/90°/90°/0°]) with high modulus ratios under SD-SD boundary conditions are considered. With different shell theory, the presented results agree well with the solutions in the represented literature by Qatu [13].

**Figure 2:**
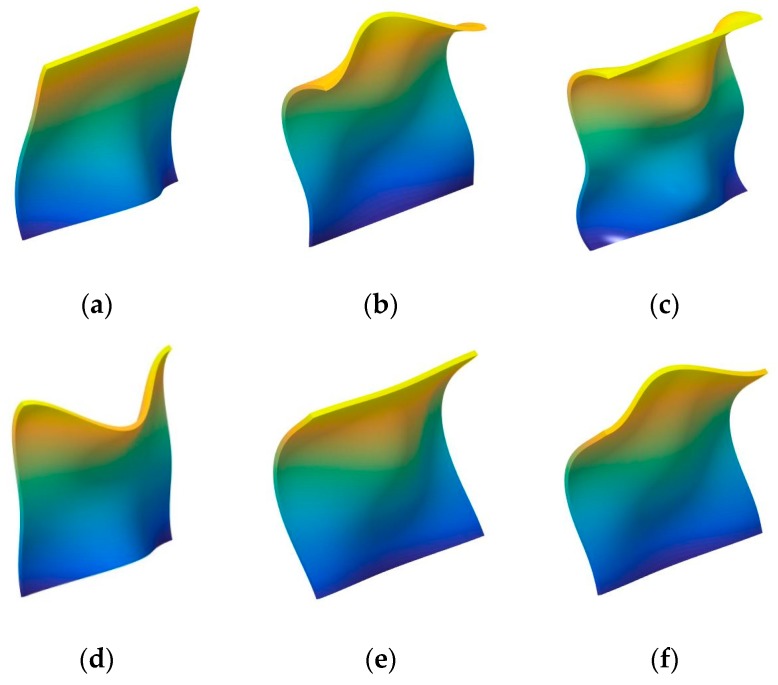

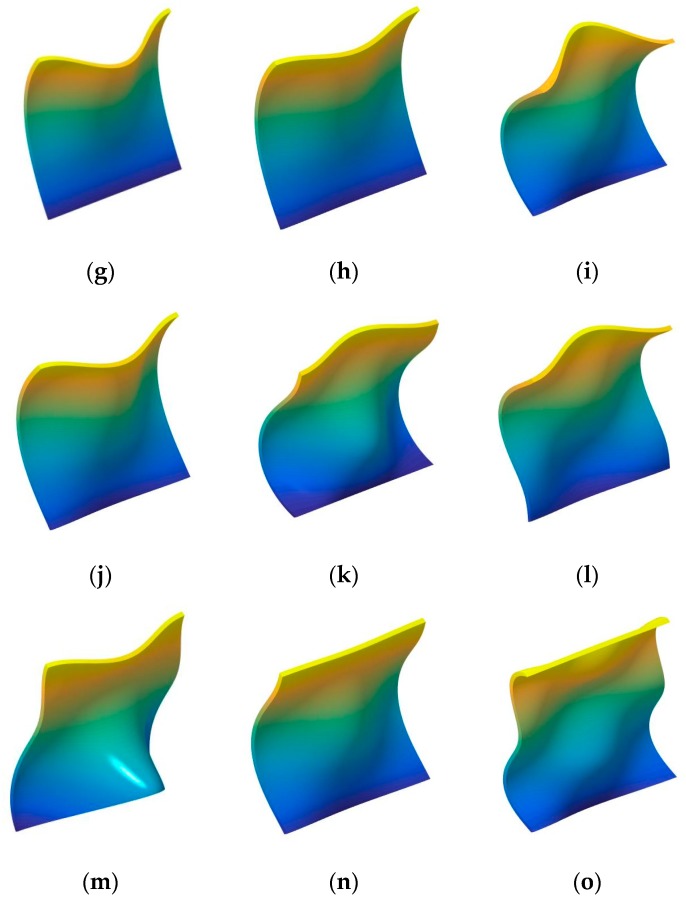
The mode shapes for the composite laminated shallow cylindrical shell with various boundary conditions. (**a**) C-C,(1,1); (**b**) C-C,(1,2); (**c**) C-C,(1,3); (**d**) F-C,(1,1); (**e**) F-C,(1,2); (**f**) F-C,(1,3); (**g**) F-F,(1,1); (**h**) F-F,(1,2); (**i**) F-F,(1,3); (**j**) F-S,(1,1); (**k**) F-S,(1,2); (**l**) F-S,(1,3); (**m**) S-C,(1,1); (**n**) S-C,(1,2); (**o**) S-C,(1,3).

**Figure 3:**
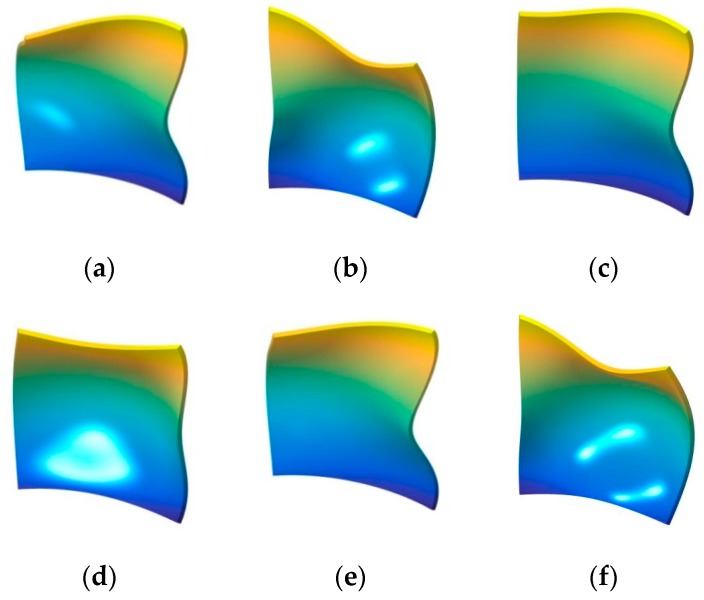

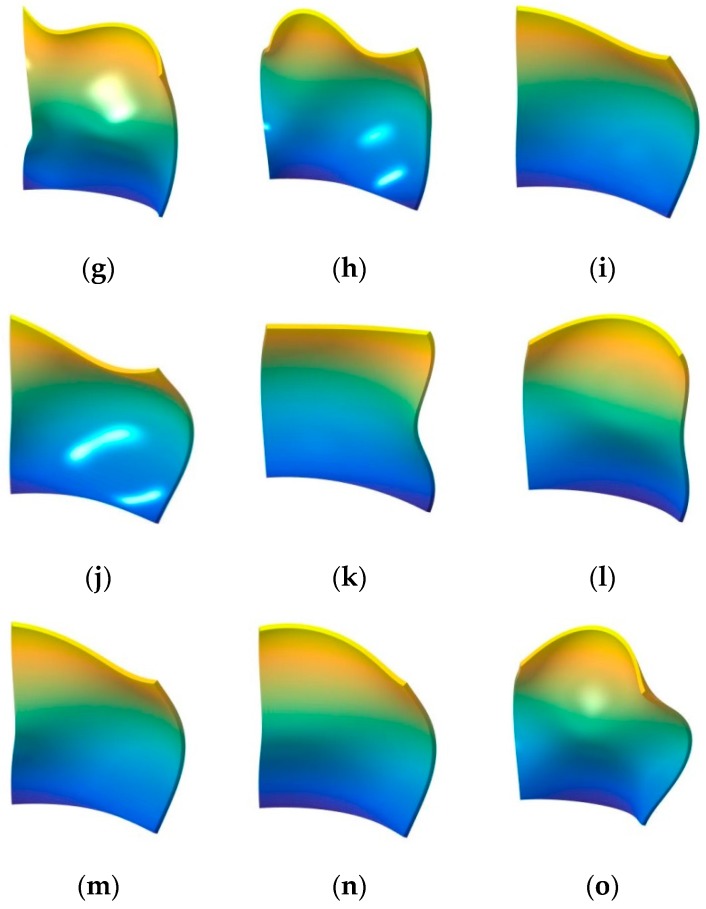
The mode shapes for the composite laminated shallow spherical shell with various boundary conditions. (**a**) C-C,(1,1); (**b**) C-C,(1,2); (**c**) C-C,(1,3); (**d**) F-C,(1,1); (**e**) F-C,(1,2); (**f**) F-C,(1,3); (**g**) F-F,(1,1); (**h**) F-F,(1,2); (**i**) F-F,(1,3); (**j**) F-S,(1,1); (**k**) F-S,(1,2); (**l**) F-S,(1,3); (**m**) S-C,(1,1); (**n**) S-C,(1,2); (**o**) S-C,(1,3).

In the next part, the fundamental frequency parameter Ω of the composite laminated shallow shell under classical combination boundary conditions is discussed. In Table 5 and Table 6, the composite laminated shallow cylindrical shell and spherical shell with various classical combination boundary conditions (i.e., F-F, F-S, F-C, S-S, S-C, C-C) are investigated by FSDT and CST. Two types of layered lamination schemes (i.e., [0°/90°] and [0°/90°/0°]) and radius constants (i.e., *R* = 5, 20) are discussed. The material constants and geometric parameters are defined as follows: *L_x_* = 1 m, *L_y_*/*L_x_* = 1, *E*_2_ = 7 GPa, *E*_1_/*E*_2_ = 25, *G*_12_ = *G*_13_ = 0.5*E*_2_, *G*_23_ = 0.2*E*_2_, *μ*_12_ = 0.25, *ρ* = 1650 kg/m^3^. Also, the fundamental frequency parameters Ω are compared with the solutions in the represented literature by Qatu [13]. From the comparison of the results by the presented method and represented literature, it can be seen that the errors obtained by the two different methods are small. The maximum error of 3.62% appears in the situation with [0°/90°/0°] composite laminated cylindrical shell (FSDT, *R* = 5) with F-F boundary condition in Table 5. Furthermore, the maximum error is 3.71% in Table 6 for the [0°/90°] composite laminated shallow spherical shell (CST, *R* = 20) with the F-F boundary condition. For various boundary conditions, the maximum parameters Ω appear when the composite shells have the C-C boundary condition. Simultaneously, the minimum frequency parameters emerge with F-F for several lamination schemes and shell theory. So, the composite laminated shallow shells with arbitrary classical combination boundary conditions by WBM can be verified through the presented numerical examples. In order to further investigate the free vibration characteristics of composite laminated shallow shells with arbitrary combination boundary conditions, some mode shapes (*n*, *m*) of the composite laminated cylindrical shell and spherical shell are shown in Figure 2 and Figure 3, respectively.

**Figure 4:**
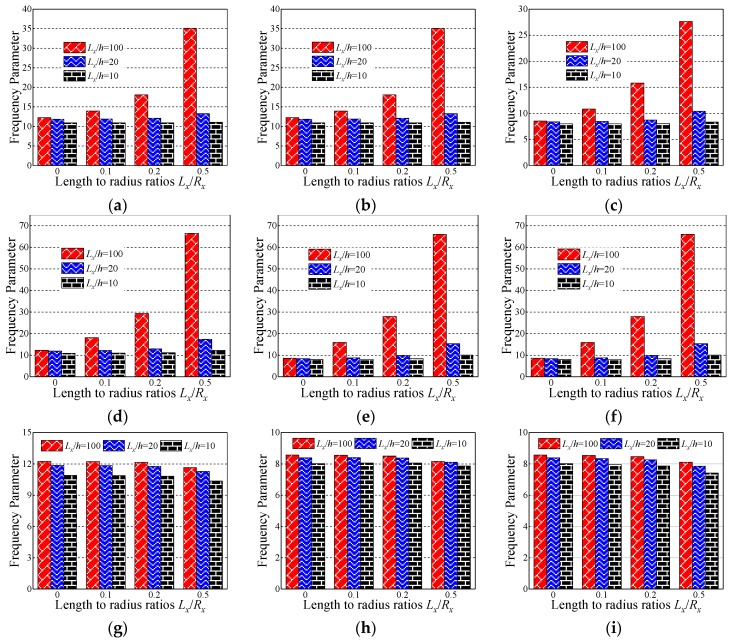
Variation laws of the fundamental frequency parameter Ω for composite laminated shallow shells with the SD-SD boundary condition with respect to various length to radius ratios *L_x_*/*R_x_* and length to thickness ratios *L_x_*/*h* by first-order shear deformation shell theory (FSDT). (**a**) Cylindrical shell, 0°/90°/90°/0°; (**b**) cylindrical shell, 0°/90°; (**c**) cylindrical shell, 90°/0°; (**d**) spherical shell, 0°/90°/90°/0°; (**e**) spherical shell, 0°/90°; (**f**) spherical shell, 90°/0°; (**g**) hyperbolic paraboloidal, 0°/90°/90°/0°; (**h**) hyperbolic paraboloidal, 0°/90°; (**i**) hyperbolic paraboloidal, 90°/0°.

**Figure 5:**
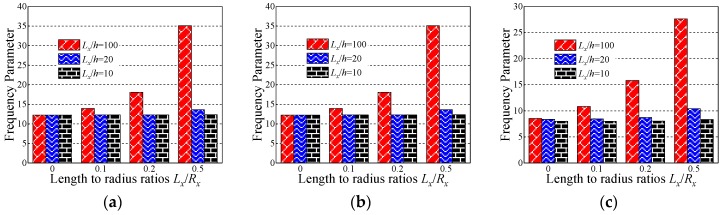

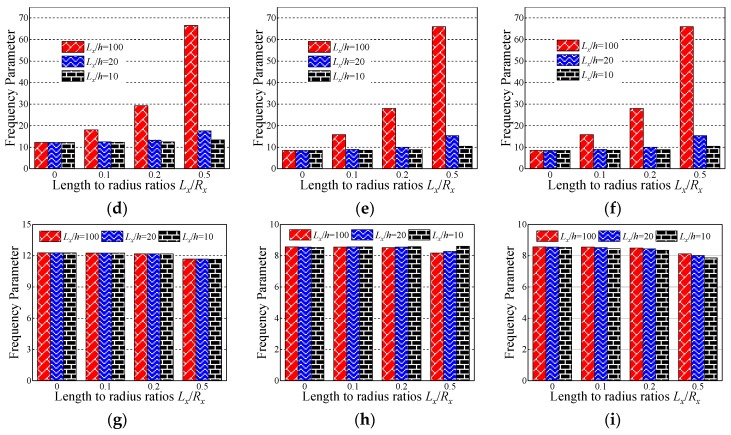
Variation laws of the fundamental frequency parameter Ω for composite laminated shallow shells with the SD-SD boundary conditions with respect to various length to radius ratios *L_x_*/*R_x_* and length to thickness ratios *L_x_*/*h* by classical shell theory (CST). (**a**) Cylindrical shell, 0°/90°/90°/0°; (**b**) cylindrical shell, 0°/90°; (**c**) cylindrical shell, 90°/0°; (**d**) spherical shell, 0°/90°/90°/0°; (**e**) spherical shell, 0°/90°; (**f**) spherical shell, 90°/0°; (**g**) hyperbolic paraboloidal, 0°/90°/90°/0°; (**h**) hyperbolic paraboloidal, 0°/90°; (**i**) hyperbolic paraboloidal, 90°/0°.

In this section, the influence of the length to thickness ratio *L_x_*/*h* and length to radius ratio *L_x_*/*R_x_* on the fundamental frequency parameter Ω is discussed. In Table 7, Table 8 and Table 9, the fundamental frequency parameter Ω for three types of the layered (i.e., [0°/90°/90°/0°], [0°/90°], and [90°/0°]) composite laminated shallow cylindrical shell, spherical shell, and hyperbolic paraboloidal shell with SD-SD by FSDT and CST is discussed. The material parameters and geometric constants are defined as follows: *L_x_* = 1 m, *L_y_*/*L_x_* = 1, *E*_2_ = 7 GPa, *E*_1_/*E*_2_ = 15, *G*_12_ = *G*_13_ = 0.5*E*_2_, *G*_23_ = 0.5*E*_2_, *μ*_12_ = 0.25, *ρ* = 1650 kg/m^3^. Especially with the composite laminated shallow shells with *L_x_*/*R_x_* = 0, the composite shells are transformed into the plate form. From Table 7, Table 8 and Table 9, with the growing of length to radius ratio *L_x_*/*R_x_* (i.e., *L_x_*/*R_x_* = 0, 0.1, 0.2, and 0.5), the fundamental frequency parameters of the composite cylindrical and spherical shell generally grow for various length to thickness ratios *L_x_*/*h*, lamination schemes, and shell theories. Simultaneously, the fundamental frequency parameters of the composite laminated hyperbolic paraboloidal shell are generally decreased with the changing of the length to radius ratio *L_x_*/*R_x_*. It can be clearly seen that, for different laminated schemes and shell theories, when the length to thickness ratio *L_x_*/*h* = 0.01, the fundamental frequency parameter of various composite laminated shallow shells increases significantly. Relatively, when *L_x_*/*h* = 0.1, the frequency parameter increases a little and remains within a stable range. To further investigate the effect of the length to thickness ratio *L_x_*/*h* on frequency parameters Ω of the composite shallow shell, the variations of the frequency parameter Ω for composite shells with SD-SD boundary conditions, with respect to diverse length to radius ratios *L_x_*/*R_x_* and length to radius ratios *L_x_*/*h*, by FSDT and CST are shown in Figure 4 and Figure 5. It can be seen that, for different laminated schemes, shallow shell structures, and shell theories, as the length to thickness ratio *L_x_*/*h* increases, the fundamental frequency parameters Ω gradually decrease. At the same time, it can be seen that, for the composite laminated hyperbolic paraboloidal shell, the variation of the fundamental frequency parameters Ω is small and the effect of the length to thickness ratio *L_x_*/*h* is not particularly obvious.

**Figure 6:**
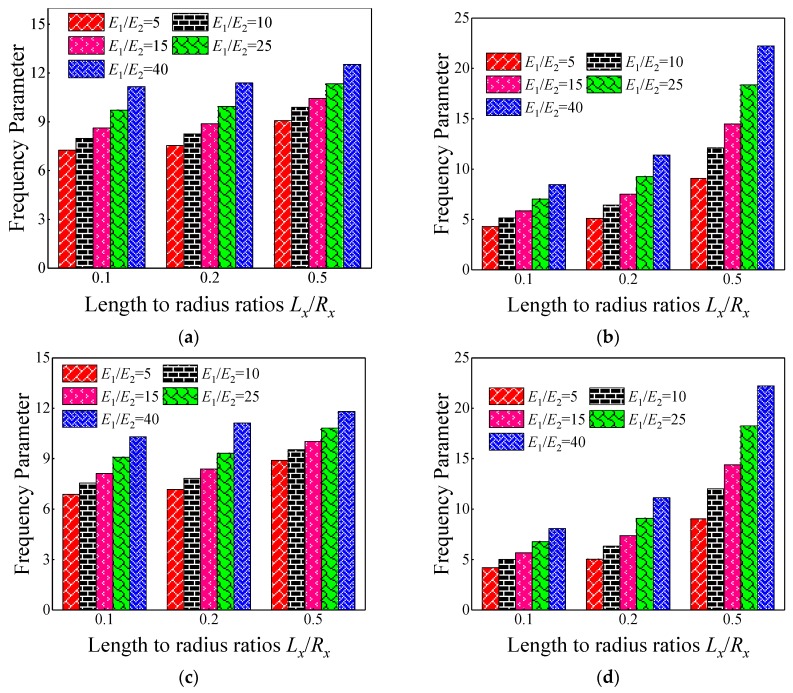
Variation laws of the fundamental frequency parameter Ω for composite laminated shallow spherical shells with various boundary conditions with respect to diverse length to radius ratios *L_x_*/*R_x_* and modulus ratios *E*_1_*/E*_2_ by FSDT and CST. (**a**) FSDT: SD-SD; (**b**) FSDT: C-C; (**c**) CST: SD-SD; (**d**) CST: C-C.

In the previous numerical example, the effect of geometric parameters on the fundamental frequency parameters is discussed. In this part, the influence of the material parameter on the frequency parameter is investigated. In Table 10 and Table 11, the fundamental frequency parameter for composite laminated shallow spherical shells with various length to radius ratios *L_x_*/*R_x_*, modulus ratios *E*_1_/*E*_2_, and boundary conditions (i.e., SD-SD, F-F, and C-C) are discussed by FSDT and CST. The material parameters and geometric constants are given as follows: *L_x_* = 1 m, *L_y_*/*L_x_* = 1, *h*/*L_y_* = 0.1, *E*_2_ = 7 GPa, *G*_12_ = *G*_13_ = 0.5*E*_2_, *G*_23_ = 0.5*E*_2_, *μ*_12_ = 0.25, *ρ* = 1650 kg/m^3^. It can be clearly seen from Table 10 and Table 11 that the fundamental frequency parameter Ω generally grows with the changing of modulus ratios *E*_1_/*E*_2_ from 5 to 40. To further reflect the impact of modulus ratios *E*_1_/*E*_2_ on fundamental frequency parameters, the variations of the fundamental frequency parameter Ω for composite spherical shells with various boundary conditions with respect to multiple length to radius ratios *L_x_*/*R_x_* and modulus ratios *E*_1_*/E*_2_ by FSDT and CST are shown in Figure 6. Therefore, it can be concluded that the modulus ratios *E*_1_*/E*_2_ has a significant effect on the fundamental frequency parameters of the composite spherical shell and plays a positive role. Different boundary conditions cause the stiffness matrix to change. For the free boundary condition, the determinant of the stiffness matrix increases with respect to the clamped boundary condition, and when the mass matrix remains unchanged, the natural frequency increases.

### 3.2. Composite Laminated Shallow Shell with Elastic Boundary Conditions

The composite laminated shallow shell with elastic constraint is widely encountered in many engineering applications. So, analysis of the composite shallow shells with such an elastic boundary condition is necessary and significant. Therefore, in this section, the free vibration characteristics of the composite shallow shell with elastic boundary conditions are discussed. 

In this section, the effect of the restrained springs on the frequency parameter of the certain cross-ply composite laminated shallow shells is discussed. The certain cross-ply layered [0°/90°] composite laminated shallow shells with S-elastic boundary conditions are concerned by FSDT. For the elastic restrained edge, there is only one set of spring component on one displacement or transverse rotational direction and the range of stiffness constants is defined as 10^0^–10^12^. The material parameters and geometric constants are defined as follows: *L_x_* = 1 m, *L_y_*/*L_x_* = 1, *R_x_*/*L_y_*, *E*_2_ = 7 GPa, *E*_1_/*E*_2_ = 25, *G*_12_ = *G*_13_ = 0.5*E*_2_, *G*_23_ = 0.2*E*_2_, *μ*_12_ = 0.25, *ρ* = 1650 kg/m^3^. In Table 12, Table 13, Table 14, Table 15 and Table 16, the lowest two frequency parameter Ω for the composite shells with S-elastic boundary conditions by restrained spring components *K_u_*, *K_v_*, *K_w_*, *K_ϕx_*, and *K_ϕy_* for a certain circumferential number of *n* = 1 is calculated.

**Figure 7:**
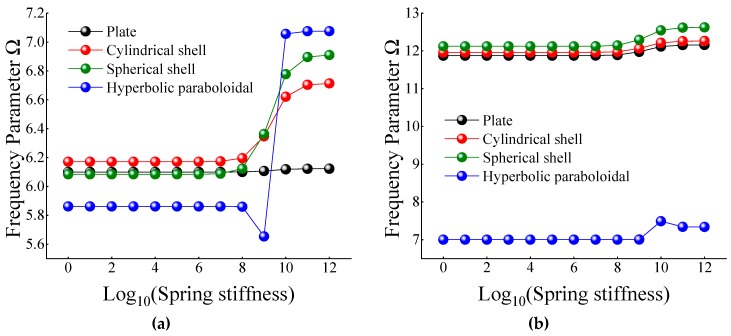
Variation laws of the frequency parameter Ω for composite shallow [0°/90°] shells with various stiffness constant *K_u_* by FSDT. (**a**) *m* = 1; (**b**) *m* = 2.

**Figure 8:**
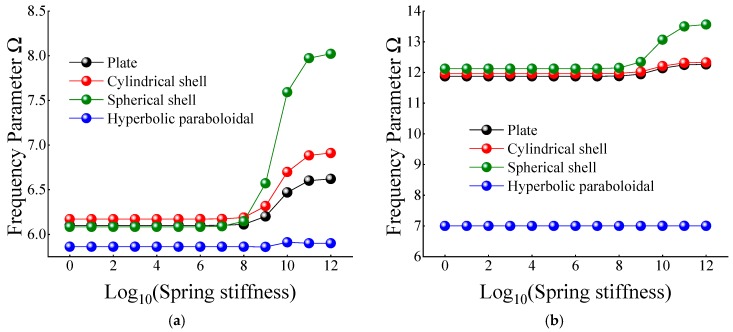
Variation laws of the frequency parameter Ω for composite shallow [0°/90°] shells with various stiffness constant *K_v_* by FSDT. (**a**) *m* = 1; (**b**) *m* = 2.

**Figure 9:**
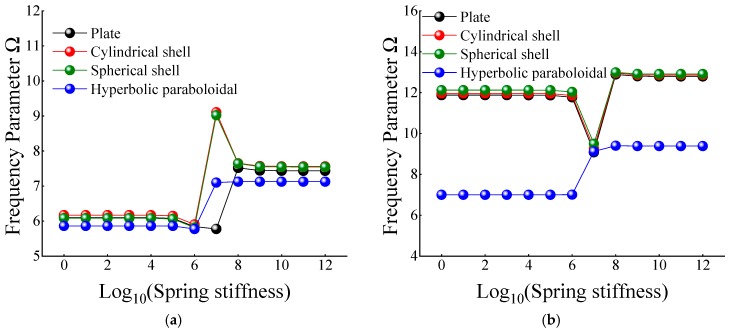
Variation laws of the frequency parameter Ω for composite shallow [0°/90°] shells with various stiffness constant *K_w_* by FSDT. (**a**) *m* = 1; (**b**) *m* = 2.

**Figure 10:**
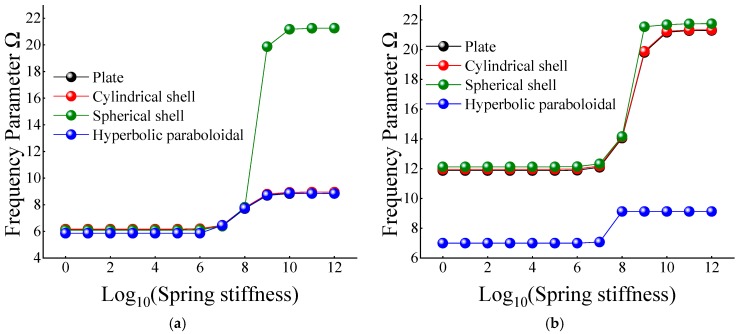
Variation laws of the frequency parameter Ω for composite shallow [0°/90°] shells with various stiffness constant *K_ϕx_* by FSDT. (**a**) *m* = 1; (**b**) *m* = 2.

**Figure 11:**
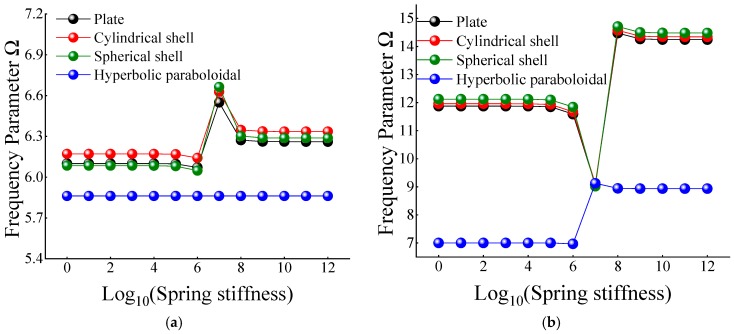
Variation laws of the frequency parameter Ω for composite shallow [0°/90°] shells with various stiffness constant *K_ϕy_* by FSDT. (**a**) *m* = 1; (**b**) *m* = 2.

In Table 12 and Table 13, when the certain cross-ply composite laminated shallow shells are only restrained in the direction of *u* and *v*, the frequency parameters generally increase with the various composite laminated shallow shell forms. Also, the increase in frequency parameters is small and basically remains within a stable range. Correspondingly, when *K_u_* = 10^6^, the frequency parameter starts to increase slightly, and when *K_u_* = 10^10^, the frequency parameter remains basically unchanged. When the composite laminated shallow shells are under the elastic restraint *K_w_* in Table 14, the frequency parameters Ω are generally decreased with the growing of the spring stiffness from 10^0^ to 10^12^. In particular, for the hyperbolic paraboloidal shell, the frequency parameter increases less than that of the other composite laminated shallow shell forms. For the effect of transverse rotational spring stiffness on the frequency parameter of composite laminated shallow shells, Table 15 and Table 16 show the changing rule of the frequency parameters with the growing of the stiffness constants for *K_ϕx_* and *K_ϕy_*. In general, as the stiffness constants *K_ϕx_* and *K_ϕy_* continue to increase, the frequency parameters corresponding to each structure tend to increase; at the same time, the main change region of the frequency parameter is between *K_ϕx,y_* = 10^4^–10^10^. However, when *K_ϕx,y_* = 10^7^, there will be some jitter in the frequency parameters, which suddenly increase or decrease. Therefore, as the elastic restrained stiffness constants in different directions increase, the frequency parameters of various composite shell forms are gradually increasing and have different change regions. Furthermore, the variations of the frequency parameter with the changing of the stiffness constants are shown in Figure 7, Figure 8, Figure 9, Figure 10 and Figure 11.

## 4. Conclusions

A semi-analyzed method is conducted for the free vibration characteristics of composite laminated shallow shells with general boundary conditions, including classical boundary conditions, elastic boundary conditions, and their combinations. Through the relationship between the displacement vector and force resultants, the formulations are established related to classical shell theory (CST) and first-order shear deformation shell theory (FSDT). According to diverse boundary conditions, the boundary matrix and the total matrix of the composite shallow shell will be established. Through the dichotomy method to search the zeros position of the total matrix determinant, the natural frequency can be obtained. Correspondingly, some numerical examples are calculated and the conclusions can be summarized as follows:

First, by comparing the solutions by the presented method with some reported literature results, the correctness of the calculation for the free vibration characteristics of composite laminated shallow shells with classical boundary conditions, elastic boundary conditions, and their combinations can be proven.

Second, some numerical examples are extended to investigate the influence of material parameters and geometric constants, like length to radius ratios, length to thickness ratios, and modulus ratios, on the frequency parameter. It can concluded that different material and geometric parameters have different influence factors on frequency parameters. Simultaneously, changing laws obtained by various composite laminated shallow shell structures are not consistent.

Finally, the effect of boundary elastic restrained stiffness on the natural frequency parameters is discussed. By changing the value of the spring stiffness in different displacement directions and transverse rotation from 10^0^ to 10^12^, the variation of the frequency parameter with the elastic restrained spring stiffness constants is obtained. It can be seen from numerical analysis examples that the different elastic constants have a positive effect on the frequency parameters and have a certain effect on the increase of the frequency parameters. Simultaneously, the effect of each spring stiffness constant has its own influence range.

## Figures and Tables

**Table 1:** The fundamental frequency parameter Ω for the composite shallow shell with the SD-SD boundary condition by first-order shear deformation shell theory (FSDT). WBM, wave-based method.

*R_x_/R_y_*	*R_y_/L_y_*	0°/90°/90°/0°				
		WBM	Ref. [13]	Error	Ref. [22]	Error
1	2	12.3093	12.5718	−2.09%	12.3633	−0.44%
	5	11.1495	11.2522	−0.91%	11.2135	−0.57%
	10	10.9672	11.0428	−0.69%	11.0329	−0.60%
0	2	11.2142	11.3342	−1.06%	11.2756	−0.54%
	5	10.9562	11.0316	−0.68%	11.0217	−0.59%
	10	10.9562	10.9867	−0.28%	10.9842	−0.25%
−1	2	10.3671	10.7031	−3.14%	10.4300	−0.60%
	5	10.8169	10.9273	−1.01%	10.8826	−0.60%
	10	10.8831	10.9605	−0.71%	10.9493	−0.60%
*R_x_/R_y_*	*R_y_/L_y_*	0°/90°				
1	2	10.0265	10.2492	−2.17%	10.0998	−0.73%
	5	8.3845	8.5084	−1.46%	8.4783	−1.11%
	10	8.1132	8.2190	−1.29%	8.2111	−1.19%
0	2	8.6166	8.7523	−1.55%	8.7075	−1.04%
	5	8.1475	8.2445	−1.18%	8.2458	−1.19%
	10	8.0644	8.1592	−1.16%	8.1636	−1.22%
−1	2	7.8626	8.0831	−2.73%	7.9596	−1.22%
	5	8.0549	8.1538	−1.21%	8.1546	−1.22%
	10	8.0537	8.1448	−1.12%	8.1534	−1.22%
*R_x_/R_y_*	*R_y_/L_y_*	90°/0°				
1	2	10.0261	10.2492	−2.18%	10.0998	−0.73%
	5	8.3843	8.5084	−1.46%	8.4783	−1.11%
	10	8.1131	8.2190	−1.29%	8.2111	−1.19%
0	2	8.3533	8.5784	−2.62%	8.4421	−1.05%
	5	8.1474	8.1774	−0.37%	8.1448	0.03%
	10	8.0644	8.1259	−0.76%	8.1136	−0.61%
−1	2	7.4152	7.7739	−4.61%	7.5071	−1.22%
	5	7.8606	8.0223	−2.02%	7.9581	−1.22%
	10	7.9554	8.0785	−1.52%	8.0540	−1.22%

**Table 2:** The fundamental frequency parameter Ω for composite shallow shell with the SD-SD boundary condition by classical shell theory (CST) theory.

*R_x_/R_y_*	*R_y_/L_y_*	0°/90°/90°/0°				
		WBM	Ref. [13]	Error	Ref. [22]	Error
1	2	66.52832	66.5774	−0.07%	66.5285	0.00%
	5	29.29062	29.309	−0.06%	29.2906	0.00%
	10	18.12154	18.129	−0.04%	18.1215	0.00%
0	2	35.10566	35.1838	−0.22%	35.1622	−0.16%
	5	18.08579	18.1107	−0.14%	18.1038	−0.10%
	10	13.96209	13.9703	−0.06%	13.9681	−0.04%
−1	2	11.67421	11.9776	−2.60%	11.6742	0.00%
	5	12.1784	12.2279	−0.41%	12.1783	0.00%
	10	12.25251	12.2649	−0.10%	12.2525	0.00%
*R_x_/R_y_*	*R_y_/L_y_*	0°/90°				
1	2	65.98726	66.0139	−0.04%	65.98717	0.00%
	5	27.95602	27.9666	−0.04%	27.95599	0.00%
	10	15.85265	15.8573	−0.03%	15.85258	0.00%
0	2	28.16678	28.2471	−0.28%	28.16667	0.00%
	5	15.84446	15.8484	−0.02%	15.81926	0.16%
	10	10.85982	10.8616	−0.02%	10.85171	0.07%
−1	2	8.121357	8.37737	−3.06%	8.17396	−0.64%
	5	8.487776	8.54161	−0.63%	8.51081	−0.27%
	10	8.545494	8.56847	−0.27%	8.55691	−0.13%
*R_x_/R_y_*	*R_y_/L_y_*	90°/0°				
1	2	65.98726	66.0139	−0.04%	66.0139	−0.04%
	5	27.95602	27.9666	−0.04%	27.9666	−0.04%
	10	15.85265	15.8573	−0.03%	15.8573	−0.03%
0	2	27.692	27.827	−0.49%	27.69195	0.00%
	5	15.81913	15.8342	−0.10%	15.84456	−0.16%
	10	10.85163	10.8567	−0.05%	10.85977	−0.08%
−1	2	8.173862	8.34143	−2.05%	8.12143	0.64%
	5	8.510863	8.52632	−0.18%	8.48796	0.27%
	10	8.557038	8.55594	0.01%	8.54535	0.14%

**Table 3:** The fundamental frequency parameter Ω for a composite plate with the SD-SD boundary condition with variety theory.

Lamination Theory								
*L_y_*/*h*	0°/90°						0°/90°/90°/0°	
	FSDT		CST		FSDT		CST	
	Ref. [13]	WBM	Ref. [13]	WBM	Ref. [13]	WBM	Ref. [13]	WBM
100	8.56394	8.55196	8.56847	8.56858	12.26147	12.26147	12.37733	12.27746
20	8.44807	8.44807	8.55811	8.55808	11.90100	11.90100	12.27733	12.27731
10	8.11956	8.11956	8.52569	8.52570	10.97163	10.97163	12.27733	12.27733
5	7.14661	7.14661	8.39526	8.39527	8.77840	8.77841	12.27733	12.27734

**Table 4:** The fundamental frequency parameter Ω for a composite plate with the SD-SD boundary condition by variety theory.

Lamination Theory				
*L_y_*//*h*	0°/90°			
	FSDT		CST	
	Ref. [13]	WBM	Ref. [13]	WBM
100	9.6873	9.6873	9.696	9.6961
10	8.9001	8.9001	9.6436	9.6436
*L_y_*//*h*	0°/90°/0°			
100	15.183	15.1834	15.228	15.2278
10	12.163	12.1629	15.228	15.2278
*L_y_*//*h*	0°/90°/90°/0°			
100	15.184	15.1839	15.228	15.2278
10	12.226	12.2272	15.228	15.2278

**Table 5:** The fundamental frequency parameter Ω for two types of the layered composite shallow cylindrical shell with various boundary conditions, theories, and radii.

Lamination Schemes	Theory	*R*		Boundary Conditions					
				F-F	F-S	F-C	S-S	S-C	C-C
0°/90°	CST	20	Ref. [13]	6.128	6.489	7.008	9.56	12.136	15.757
			WBM	6.147	6.376	7.257	9.633	12.236	15.895
			Error	0.31%	−1.74%	3.55%	0.77%	0.82%	0.88%
		5	Ref. [13]	6.096	6.444	7.014	9.598	12.154	15.747
			WBM	6.070	6.433	7.231	9.630	12.225	15.861
			Error	−0.42%	−0.16%	3.10%	0.34%	0.58%	0.72%
	FSDT	20	Ref. [13]	5.763	6.087	6.535	8.894	10.609	12.623
			WBM	5.778	6.007	6.532	8.803	10.555	12.621
			Error	0.26%	−1.32%	−0.05%	−1.03%	−0.50%	−0.01%
		5	Ref. [13]	5.716	6.030	6.524	8.931	10.647	12.663
			WBM	5.850	5.994	6.502	8.826	10.582	12.651
			Error	2.34%	−0.59%	−0.34%	−1.17%	−0.61%	−0.10%
0°/90°/0°	CST	20	Ref. [13]	3.902	4.484	6.866	15.106	22.557	32.091
			WBM	3.966	4.501	6.891	15.229	22.214	32.385
			Error	1.63%	0.39%	0.36%	0.82%	−1.52%	0.92%
		5	Ref. [13]	3.894	4.472	6.901	15.136	22.560	32.062
			WBM	3.966	4.475	6.917	15.253	22.214	32.352
			Error	1.84%	0.07%	0.23%	0.77%	−1.53%	0.90%
	FSDT	20	Ref. [13]	3.787	4.318	6.146	12.166	14.250	16.385
			WBM	3.796	4.312	6.146	12.104	14.218	16.384
			Error	0.23%	−0.14%	0.00%	−0.51%	−0.22%	0.00%
		5	Ref. [13]	3.773	4.301	6.176	12.212	14.284	16.408
			WBM	3.910	4.392	6.170	12.148	14.250	16.406
			Error	3.62%	2.11%	−0.09%	−0.53%	−0.24%	−0.01%

**Table 6:** The fundamental frequency parameter Ω for two types of the layered composite shallow spherical shell with various boundary conditions, theories, and radii.

Lamination Schemes	Theory	*R*		Boundary Conditions					
				F-F	F-S	F-C	S-S	S-C	C-C
0°/90°	CST	20	Ref. [13]	6.132	6.493	7.002	9.588	12.165	15.822
			WBM	6.360	6.360	7.066	9.663	12.274	15.975
			Error	3.71%	−2.05%	0.91%	0.78%	0.90%	0.97%
		5	Ref. [13]	6.162	6.51	6.971	9.903	12.465	16.82
			WBM	6.250	6.482	6.940	9.945	12.560	16.990
			Error	1.43%	−0.43%	−0.44%	0.42%	0.77%	1.01%
	FSDT	20	Ref. [13]	5.768	6.093	6.535	8.922	10.64	12.713
			WBM	5.764	6.076	6.532	8.833	10.590	12.714
			Error	−0.07%	−0.27%	−0.04%	−1.00%	−0.47%	0.01%
		5	Ref. [13]	5.787	6.105	6.511	9.247	11.004	14.081
			WBM	5.765	6.073	6.493	9.146	10.946	14.078
			Error	−0.38%	−0.53%	−0.27%	−1.09%	−0.53%	−0.02%
0°/90°/0°	CST	20	Ref. [13]	3.909	4.49	6.863	15.116	22.562	32.136
			WBM	3.924	4.512	6.888	15.236	22.214	32.430
			Error	0.38%	0.49%	0.36%	0.79%	−1.54%	0.91%
		5	Ref. [13]	4.009	4.562	6.861	15.29	22.64	32.785
			WBM	4.010	4.599	6.909	15.356	22.215	33.051
			Error	0.02%	0.81%	0.70%	0.43%	−1.88%	0.81%
	FSDT	20	Ref. [13]	3.794	4.325	6.146	12.178	14.264	16.487
			WBM	3.794	4.319	6.146	12.114	14.231	16.486
			Error	0.00%	−0.14%	−0.01%	−0.52%	−0.23%	−0.01%
		5	Ref. [13]	3.891	4.397	6.163	12.394	14.499	17.959
			WBM	3.884	4.369	6.163	12.312	14.454	17.951
			Error	−0.18%	−0.63%	0.00%	−0.66%	−0.31%	−0.04%

**Table 7:** The fundamental frequency parameter Ω for three types of layered composite shallow cylindrical shells for variety theories, length to thickness ratios *L_x_*/*h*, and length to radius ratios *L_x_*/*R_x_* with the SD-SD boundary condition.

*L_x_/h*	*L_x_/R_x_*	Lamination Schemes					
		0°/90°/90°/0°		0°/90°		90°/0°	
		FSDT	CST	FSDT	CST	FSDT	CST
100	0	12.2531	12.2775	8.5520	8.5686	8.5520	8.5686
	0.1	13.9407	13.9621	10.8462	10.8598	10.8382	10.8516
	0.2	18.0687	18.0858	15.8342	15.8445	15.8090	15.8192
	0.5	35.0954	35.1057	28.1209	28.1668	27.6433	27.6921
20	0	11.8617	12.2773	8.3908	8.5581	8.3908	8.5581
	0.1	11.9222	12.3350	8.5016	8.6680	8.4735	8.6386
	0.2	12.1011	12.2811	8.8000	8.9626	8.7410	8.9010
	0.5	13.2636	13.6185	10.6002	10.7427	10.4142	10.5501
10	0	10.9053	12.2773	8.0202	8.5257	8.0202	8.5257
	0.1	10.9125	12.2803	8.0644	8.5724	8.0149	8.5146
	0.2	10.9338	12.2894	8.1475	8.6547	8.0476	8.5383
	0.5	11.0809	12.3534	8.6166	9.1042	8.3533	8.8006

**Table 8:** The fundamental frequency parameter Ω for three types of layered composite shallow spherical shells for variety theories, length to thickness ratios *L_x_*/*h*, and length to radius ratios *L_x_*/*R_x_* with the SD-SD boundary condition.

*L_x_/h*	*L_x_/R_x_*	Lamination Schemes					
		0°/90°/90°/0°		0°/90°		90°/0°	
		FSDT	CST	FSDT	CST	FSDT	CST
100	0	12.2531	12.2775	8.5520	8.5686	8.5520	8.5686
	0.1	18.1044	18.1215	15.8423	15.8526	15.8423	15.8526
	0.2	29.2786	29.2906	27.9478	27.9560	27.9478	27.9560
	0.5	66.5196	66.5283	65.9775	65.9873	65.9775	65.9873
20	0	11.8617	12.2773	8.3908	8.5581	8.3908	8.5581
	0.1	12.1375	12.5436	8.7873	8.9489	8.7873	8.9489
	0.2	12.9248	13.3057	9.8753	10.0240	9.8753	10.0240
	0.5	17.3215	17.6007	15.3115	15.4258	15.3114	15.4258
10	0	10.9053	12.2773	8.0202	8.5257	8.0202	8.5257
	0.1	10.9672	12.3284	8.1132	8.6132	8.1131	8.6132
	0.2	11.1495	12.4794	8.3845	8.8692	8.3843	8.8692
	0.5	12.3093	13.4531	10.0265	10.4333	10.0261	10.4333

**Table 9:** The fundamental frequency parameter Ω for three types of layered composite shallow hyperbolic paraboloidal shells for variety theories, length to thickness ratios *L_x_*/*h*, and length to radius ratios *L_x_*/*R_x_* with the SD-SD boundary condition.

*L_x_/h*	*L_x_/R_x_*	Lamination Schemes					
		0°/90°/90°/0°		0°/90°		90°/0°	
		FSDT	CST	FSDT	CST	FSDT	CST
100	0	12.2531	12.2775	8.5520	8.5686	8.5520	8.5686
	0.1	12.2283	12.2525	8.5404	8.5570	8.5289	8.5454
	0.2	12.1543	12.1783	8.4944	8.5109	8.4716	8.4877
	0.5	11.6512	11.6742	8.1583	8.1739	8.1059	8.1213
20	0	11.8617	12.2773	8.3908	8.5581	8.3908	8.5581
	0.1	11.8377	12.2524	8.4015	8.5698	8.3464	8.5120
	0.2	11.7659	12.1778	8.3783	8.5466	8.2693	8.4325
	0.5	11.2784	11.6712	8.1079	8.2715	7.8571	8.0092
10	0	10.9053	12.2773	8.0202	8.5257	8.0202	8.5257
	0.1	10.8831	12.2519	8.0537	8.5664	7.9554	8.4514
	0.2	10.8169	12.1761	8.0549	8.5724	7.8606	8.3452
	0.5	10.3671	11.6619	7.8626	8.6024	7.4152	7.8559

**Table 10:** The fundamental frequency parameter Ω for composite shallow [0°/90°] spherical shells for variety length to radius ratios *L_x_*/*R_x_*, modulus ratios *E*_1_/*E*_2_, and boundary conditions by FSDT.

Boundary Conditions	*L_x_*/*R_x_*	*E*_1_/*E*_2_				
		5	10	15	25	40
SD-SD	0.1	6.8768	7.5476	8.1132	9.0869	10.3019
	0.2	7.1668	7.8326	8.3845	9.3295	11.1241
	0.5	8.8937	9.5415	10.0265	10.8234	11.8112
F-F	0.1	3.9347	4.5645	5.0554	5.8596	6.8237
	0.2	3.9482	4.5782	5.0664	5.8626	6.8150
	0.5	4.0430	4.6793	5.1581	5.9229	6.8248
C-C	0.1	4.2046	5.0239	5.6852	6.7780	8.0950
	0.2	5.0485	6.3327	7.3725	9.0778	11.1241
	0.5	9.0303	12.0219	14.4032	18.2531	22.2144

**Table 11:** The fundamental frequency parameter Ω for composite shallow [0°/90°] spherical shells for variety length to radius ratios *L_x_*/*R_x_*, modulus ratios *E*_1_/*E*_2_, and boundary conditions by CST.

Boundary Conditions	*L_x_*/*R_x_*	*E*_1_/*E*_2_				
		5	10	15	25	40
SD-SD	0.1	7.2637	7.9884	8.6201	9.7202	11.1634
	0.2	7.5404	8.2592	8.8759	9.9450	11.3972
	0.5	9.0807	9.8983	10.4389	11.3430	12.5272
C-C	0.1	4.2891	5.1468	5.8463	7.0198	8.4698
	0.2	5.1199	6.4318	7.4987	9.2602	11.3972
	0.5	9.0807	12.0872	14.4816	18.3556	22.2167

**Table 12:** The frequency parameter Ω for composite shallow [0°/90°] shells with S-*K_u_* boundary conditions by FSDT.

Stiffness	Plate		Cylindrical Shell		Spherical Shell		Hyperbolic Paraboloidal	
	*m* = 1	*m* = 2	*m* = 1	*m* = 2	*m* = 1	*m* = 2	*m* = 1	*m* = 2
10^0^	6.0999	11.8764	6.1713	11.9613	6.0843	12.1225	5.8619	7.0016
10^1^	6.0999	11.8764	6.1713	11.9613	6.0843	12.1225	5.8619	7.0016
10^2^	6.0999	11.8764	6.1713	11.9613	6.0843	12.1225	5.8619	7.0016
10^3^	6.0999	11.8764	6.1713	11.9613	6.0843	12.1225	5.8619	7.0016
10^4^	6.0999	11.8764	6.1713	11.9613	6.0843	12.1225	5.8619	7.0016
10^5^	6.0999	11.8764	6.1713	11.9614	6.0843	12.1226	5.8619	7.0016
10^6^	6.0999	11.8766	6.1716	11.9615	6.0847	12.1228	5.8619	7.0016
10^7^	6.1000	11.8780	6.1739	11.9629	6.0884	12.1251	5.8619	7.0016
10^8^	6.1009	11.8911	6.1960	11.9763	6.1241	12.1468	5.8600	7.0017
10^9^	6.1070	11.9770	6.3472	12.0653	6.3634	12.2935	5.6539	7.0042
10^10^	6.1187	12.1169	6.6216	12.2166	6.7778	12.5456	7.0567	7.4902
10^11^	6.1223	12.1553	6.7039	12.2594	6.8965	12.6167	7.0752	7.3410
10^12^	6.1228	12.1598	6.7137	12.2645	6.9106	12.6251	7.0754	7.3395

**Table 13:** The frequency parameter Ω for composite shallow [0°/90°] shells with S-*K_v_* boundary conditions by FSDT.

Stiffness	Plate		Cylindrical Shell		Spherical Shell		Hyperbolic Paraboloidal	
	*m* = 1	*m* = 2	*m* = 1	*m* = 2	*m* = 1	*m* = 2	*m* = 1	*m* = 2
10^0^	6.0999	11.8764	6.1713	11.9613	6.0843	12.1225	5.8619	7.0016
10^1^	6.0999	11.8764	6.1713	11.9613	6.0843	12.1225	5.8619	7.0016
10^2^	6.0999	11.8764	6.1713	11.9613	6.0843	12.1225	5.8619	7.0016
10^3^	6.0999	11.8764	6.1713	11.9613	6.0843	12.1225	5.8619	7.0016
10^4^	6.0999	11.8764	6.1713	11.9613	6.0843	12.1225	5.8619	7.0016
10^5^	6.0999	11.8764	6.1713	11.9613	6.0844	12.1226	5.8619	7.0016
10^6^	6.1000	11.8765	6.1715	11.9614	6.0849	12.1228	5.8619	7.0016
10^7^	6.1012	11.8772	6.1731	11.9621	6.0907	12.1251	5.8619	7.0016
10^8^	6.1122	11.8844	6.1890	11.9686	6.1466	12.1476	5.8619	7.0016
10^9^	6.2016	11.9437	6.3170	12.0234	6.5714	12.3419	5.8600	7.0016
10^10^	6.4698	12.1403	6.6986	12.2091	7.5921	13.0667	5.9118	7.0022
10^11^	6.6018	12.2486	6.8848	12.3139	7.9735	13.4990	5.9009	7.0021
10^12^	6.6204	12.2645	6.9109	12.3294	8.0215	13.5632	5.9008	7.0021

**Table 14:** The frequency parameter Ω for composite shallow [0°/90°] shells with S-*K_w_* boundary conditions by FSDT.

Stiffness	Plate		Cylindrical Shell		Spherical Shell		Hyperbolic Paraboloidal	
	*m* = 1	*m* = 2	*m* = 1	*m* = 2	*m* = 1	*m* = 2	*m* = 1	*m* = 2
10^0^	6.0999	11.8764	6.1713	11.9613	6.0843	12.1225	5.8619	7.0016
10^1^	6.0999	11.8764	6.1713	11.9613	6.0843	12.1225	5.8619	7.0016
10^2^	6.0999	11.8764	6.1713	11.9613	6.0843	12.1225	5.8619	7.0016
10^3^	6.0999	11.8765	6.1713	11.9614	6.0843	12.1226	5.8619	7.0016
10^4^	6.1002	11.8766	6.1716	11.9616	6.0846	12.1227	5.8619	7.0016
10^5^	6.1031	11.8786	6.1745	11.9635	6.0876	12.1245	5.8619	7.0016
10^6^	6.1315	11.8980	6.2027	11.9826	6.1172	12.1424	5.8605	7.0024
10^7^	6.3925	12.0948	6.4630	12.1764	6.3902	12.3238	6.4573	7.0719
10^8^	7.7073	14.0587	7.7845	14.1180	7.8101	14.1629	7.7352	9.1254
10^9^	8.7004	19.8103	8.7935	19.8887	19.8721	21.5398	8.7320	9.1254
10^10^	8.8380	21.1601	8.9336	21.2291	21.1699	21.6778	8.8733	9.1254
10^11^	8.8521	21.2786	8.9478	21.3223	21.2528	21.7339	8.8433	9.1254
10^12^	8.8535	21.2894	8.9493	21.3296	21.2599	21.7405	8.8434	9.1254

**Table 15:** The frequency parameter Ω for composite shallow [0°/90°] shells with S-*K_ϕx_* boundary conditions by FSDT.

Stiffness	Plate		Cylindrical Shell		Spherical Shell		Hyperbolic Paraboloidal	
	*m* = 1	*m* = 2	*m* = 1	*m* = 2	*m* = 1	*m* = 2	*m* = 1	*m* = 2
10^0^	6.0999	11.8764	6.1713	11.9613	6.0843	12.1225	5.8619	7.0016
10^1^	6.0999	11.8764	6.1713	11.9613	6.0843	12.1225	5.8619	7.0016
10^2^	6.0999	11.8764	6.1713	11.9613	6.0843	12.1225	5.8619	7.0016
10^3^	6.0999	11.8762	6.1713	11.9611	6.0843	12.1223	5.8619	7.0016
10^4^	6.0997	11.8739	6.1711	11.9588	6.0840	12.1200	5.8619	7.0016
10^5^	6.0974	11.8507	6.1688	11.9354	6.0812	12.0971	5.8619	7.0013
10^6^	6.0711	11.5921	6.1418	11.6754	6.0485	11.8418	5.8619	6.9694
10^7^	6.5494	9.0772	6.6282	9.1053	6.6639	9.0215	5.8621	9.1254
10^8^	6.2722	14.4763	6.3469	14.5752	6.3025	14.7114	5.8621	8.9458
10^9^	6.2620	14.2671	6.3365	14.3650	6.2894	14.5019	5.8621	8.9351
10^10^	6.2611	14.2479	6.3356	14.3457	6.2882	14.4828	5.8621	8.9350
10^11^	6.2610	14.2460	6.3355	14.3438	6.2881	14.4808	5.8621	8.9350
10^12^	6.2609	14.2458	6.3355	14.3436	6.2880	14.4807	5.8621	8.9350

**Table 16:** The frequency parameter Ω for composite shallow [0°/90°] shells with S-*K_ϕy_* boundary conditions by FSDT.

Stiffness	Plate		Cylindrical Shell		Spherical Shell		Hyperbolic Paraboloidal	
	*m* = 1	*m* = 2	*m* = 1	*m* = 2	*m* = 1	*m* = 2	*m* = 1	*m* = 2
10^0^	6.0999	11.8764	6.1713	11.9613	6.0843	12.1225	5.8619	7.0016
10^1^	6.0999	11.8764	6.1713	11.9613	6.0843	12.1225	5.8619	7.0016
10^2^	6.0999	11.8764	6.1713	11.9613	6.0843	12.1225	5.8619	7.0016
10^3^	6.0997	11.8763	6.1711	11.9612	6.0841	12.1225	5.8619	7.0016
10^4^	6.0977	11.8755	6.1691	11.9604	6.0820	12.1217	5.8619	7.0016
10^5^	6.0780	11.8669	6.1485	11.9521	6.0611	12.1145	5.8610	7.0017
10^6^	5.8434	11.7713	5.9050	11.8600	5.8139	12.0349	5.7743	7.0070
10^7^	5.7759	9.0847	9.1053	9.2703	9.0215	9.4964	7.1004	9.1254
10^8^	7.5204	12.8896	7.6516	12.9518	7.6426	12.9930	7.1247	9.4010
10^9^	7.4432	12.8039	7.5710	12.8671	7.5546	12.9172	7.1250	9.3841
10^10^	7.4360	12.7961	7.5634	12.8594	7.5463	12.9103	7.1250	9.3840
10^11^	7.4352	12.7954	7.5627	12.8587	7.5455	12.9096	7.1250	9.3840
10^12^	7.4352	12.7953	7.5626	12.8586	7.5454	12.9095	7.1250	9.3840
